# Fitting a function to time-dependent ensemble averaged data

**DOI:** 10.1038/s41598-018-24983-y

**Published:** 2018-05-03

**Authors:** Karl Fogelmark, Michael A. Lomholt, Anders Irbäck, Tobias Ambjörnsson

**Affiliations:** 1 Computational Biology and Biological Physics, Department of Astronomy and Theoretical Physics, Lund University, 223 62 Lund, Sweden; 2 2Department of Physics, Chemistry and Pharmacy, University of Southern Denmark, Campusvej 55, 5230 Odense M, Denmark

## Abstract

Time-dependent ensemble averages, i.e., trajectory-based averages of some observable, are of importance in many fields of science. A crucial objective when interpreting such data is to fit these averages (for instance, squared displacements) with a function and extract parameters (such as diffusion constants). A commonly overlooked challenge in such function fitting procedures is that fluctuations around mean values, by construction, exhibit temporal correlations. We show that the only available general purpose function fitting methods, correlated chi-square method and the weighted least squares method (which neglects correlation), fail at either robust parameter estimation or accurate error estimation. We remedy this by deriving a new closed-form error estimation formula for weighted least square fitting. The new formula uses the full covariance matrix, i.e., rigorously includes temporal correlations, but is free of the robustness issues, inherent to the correlated chi-square method. We demonstrate its accuracy in four examples of importance in many fields: Brownian motion, damped harmonic oscillation, fractional Brownian motion and continuous time random walks. We also successfully apply our method, weighted least squares including correlation in error estimation (WLS-ICE), to particle tracking data. The WLS-ICE method is applicable to arbitrary fit functions, and we provide a publically available WLS-ICE software.

## Introduction

Time-dependent ensemble averages appear in several scientific fields. Examples include: particle tracking experiments where mean square displacements (MSD) are measured at different sampling times^1^, human travel dynamics where dispersal distance as a function of time are measured^2^, single-molecule pulling experiments^3^, applications of fluctuation theorems^4^ such as the Jarzynski equality^5^, measurements of the time-dependence of donor-acceptor distance dynamics^6^, tracer particle dynamics in complex systems^7^ and correlation functions in spin systems and lattice gauge theories^8^. The final step when interpreting ensemble averages is often to fit a function to these averages in order to extract parameters.

Fitting a function to data is done so readily in science that one seldom considers the correctness of the standard go-to solution of the (linear and non-linear) weighted least squares (WLS) method^9–11^. One of the crucial implicit assumptions of the “standard” version of this method is that the fluctuations around mean values are independent. However, since for time-dependent ensemble averages the data is sampled along trajectories, this independence assumption is in general *not* satisfied when analyzing ensemble averages; heuristically, if in one trajectory an observable, such as the square displacement, was smaller than its ensemble averaged value at some time, it is typically still so at the next time step. For an illustrative example, see Figure S1 in Supplementary Information, which shows the time-evolution in simulations of fractional Brownian motion (FBM). Thus, the fluctuations around an ensemble averaged (time-dependent) observable will in general exhibit temporal correlations. Herein, the term trajectory is used in its widest sense: an observable (such as squared displacement) is chosen, and a trajectory is then measurements of this observable at different consecutive sampling times.

The question now arises of how severe the consequences of neglecting the temporal correlations in least squares fitting are. We demonstrate that such neglect leads to unreliable error estimation for parameters and can in some cases lead to underestimated errors for fitted parameters (such as diffusion constants) by more than one order of magnitude for our prototype systems (see below). The unreliability of the estimated errors can have detrimental effects when statistically interpreting the data: The 1*σ* (2*σ*) rule for Gaussian statistics states that 68% (95%) of the observed data should (on average) fall within ±1 (±2) *σ* from the estimated mean. For this rule to be meaningful one must have a correct estimator for the variance in estimated parameters, *σ*^2^.

To our knowledge, the only previous method for dealing fully with correlation in data for function fitting to ensemble-averages is the correlated chi-square method (CCM)^12,13^. This method is known to the lattice quantum chromodynamics community, but does not seem to have found wide spread use. This could partly be due to that, while mathematically sound, numerical robustness issues have been identified^14,15^. We here carefully examine the CCM method and demonstrate that it in general only provides correct parameter estimation in a small region of the “phase space” (*N*, *M*), where *N* is the number of sampling times and *M* is the number of trajectories. Thus, it appears that the CCM is of limited general purpose use for fitting of time-dependent ensemble averages to a model function.

Although the least squares and WLS methods are common techniques for parameter estimation from ensemble averages, alternative methods exist, e.g., for inferring parameters from trajectories for biological systems^16–18^. In particular, for Brownian motion (BM) an optimal estimator for the diffusion constant has recently been derived^19–21^. Bayesian methods^11,22–26^ have also been used for parameter estimation for certain classes of systems. In general, when they apply, these methods give more precise parameter estimates than the WLS method. However, these newer approaches require as input a full stochastic model of the process, and we refer to this type of approach as *model matching* methods. By a full stochastic model we here refer to a model from which (in principle) any probability or average of a measured observable can be calculated. A simple example is BM, where the time-evolution is described by a Langevin equation with a noise term for which the statistics is fully specified. In contrast, the WLS and CCM methods are parametric *function fitting*^27^ type methods, which can be used even if a full stochastic model is not available to describe the data at hand. An example from single-particle tracking, where function fitting is useful, is if one wants to determine a power-law exponent for the scaling of the mean-square displacement with time. In this situation, a function fitting procedure such as WLS can be used, without making any assumption about the underlying dynamics. Also, even if a full stochastic model is indeed available, it might be impractical to carry out a full model matching procedure.

In this article, we derive a mathematically rigorous expression for the variance and covariance of estimated parameters in WLS fitting. Our new error estimation formula for fitted WLS parameters takes into account the temporal correlations, which are intrinsic to ensemble averages based on trajectories. To avoid confusion we term the “standard” WLS method^9–11^ (i.e., weighted least squares neglecting correlation) as WLS-ECE (Weighted Least Squares Excluding Correlation in Error estimation), whereas our new approach is referred to as WLS-ICE (Weighted Least Squares Including Correlation in Error estimation). In figures and discussion where we only consider parameter values and not the associated errors, we only use the term WLS. In contrast to the previous two methods (WLS-ECE and CCM), our new method has the desirable unique features of providing both (1) robust parameter estimates in the full phase space (*N*, *M*) with mean parameter values in agreement with theory for our prototype systems; (2) error estimates that reproduce the observed spreads in our fitted parameters.

As prototype models we use BM, damped harmonic oscillation (DHO) in a heat bath, FBM and continuous time random walks (CTRW). These have been identified as important model systems in a wide range of systems. BM is of interest to many fields of science^28–30^. Variants of DHO appear in physics, engineering and chemistry^31^. FBM has been applied, for instance, to protein dynamics^6^, in financial modeling^32^, for analyzing climate time series^33^, to describe tracer particle diffusion^7,34^ and for modeling earth quake phenomena^35^. Recent applications of CTRW^28,36^ include modeling of human travel patterns^2^ and of molecular motions in cells and cell membrane^34,37^. However, we point out that our model systems are merely convenient examples for illustrating our WLS-ICE function fitting procedure, which can be applied to arbitrary fit functions. Our four model systems provide ideal test beds for our method, because the functions to be fitted, the mean position and MSD, are known analytically for these systems. Moreover, trajectories are fast to generate for these systems, which, which facilitates stringent testing of the fitting methods based on a relatively large number of trajectories.

We finally point out two restrictions on the scope of our study: First, we do not concern ourselves with the model selection problem^11,38^, i.e., how to choose the “best” model or “best” form for the fit function. Second, in single particle tracking (one of the application fields of our results), it is common to separate between time-averaged observables (such as the time-averaged MSD) and ensemble averaged observables^39,40^. In certain cases, these averages are described by the same functional form, but this is not always so^40^. In this study our sole focus is on ensemble averaged observables.

## Methods

In what follows, we provide a ready-to-use method, which is further motivated and detailed in Section A in Supplementary Information.

### The WLS-ICE procedure

In experiments or simulations one records a set of trajectories, here denoted by *m*. The task at hand is to fit some functional form *f*(*t*_*i*_;***θ***) = *f*_*i*_(***θ***), with *K* free fitting parameters ***θ*** = *θ*_1_, …, *θ*_*K*_ to some ensemble averaged observable $\overline{y}({t}_{i})={\overline{y}}_{i}$ over the trajectories, i.e., to a sample mean of the form

1 $${\overline{y}}_{i}=\frac{1}{M}\sum _{m=1}^{M}{y}_{i}^{(m)}$$

where the index *i* is over the *N* sampling times ***T*** = *T*_1_, …, *T*_*N*_ (with $N\ge K$). Herein, we use bold symbols to denote vectors or matrices. For BM, FBM and CTRW (see Results), which are all zero mean processes, the observable used is the squared displacements, i.e., ${y}_{i}^{(m)}=|{{\boldsymbol{x}}}^{(m)}({T}_{i})-{{\boldsymbol{x}}}^{(m)}(0){|}^{2}$, where ***x***^(*m*)^(*t*) is the position (a vector with *d* components, where *d* is the number of spatial dimensions) at process time *t* for trajectory *m*, and the start time for the simulation/experiment is *t* = 0. For DHO, our non-zero-mean prototype process, we instead use the position directly as relevant observable, ${y}_{i}^{(m)}={x}^{(m)}({T}_{i})$. It is important to point out, however, that in the fitting procedure the quantity ${y}_{i}^{(m)}$ can be any observable for trajectory *m* at sampling time *T*_*i*_. We shall consistently use a ‘bar’ to denote a sample estimator (we only make use of sample means and sample covariances). The challenge in function fitting procedures^10^ is to fit some function *f*_*i*_(***θ***) to the data ${\overline{y}}_{i}$ and thereby extract the model parameters, ***θ***. This problem has previously been tackled using the WLS-ECE or CCM methods (reviewed in Section B in Supplementary Information).

Our approach, the WLS-ICE method, extends the WLS-ECE procedure with a correct error estimation formula which takes correlations in fluctuations around ensemble averages into account (see Introduction). For completeness and ease of application, we here provide the full details of the proposed WLS-ICE fitting procedure. We start by introducing a cost function, χ^2^, based on the the difference between the sample average and the fit function ${{\rm{\Lambda }}}_{i}={f}_{i}({\boldsymbol{\theta }})-{\overline{y}}_{i}$ for all time points, according to

2 $${\chi }^{2}={{\boldsymbol{\Lambda }}}^{T}{\boldsymbol{R}}{\boldsymbol{\Lambda }},$$

where ***R*** is a symmetric positive definite matrix. This cost function is to be minimized with respect to ***θ*** in order to determine the best parameter values, ${\hat{\theta }}_{a}$ (*a* = 1, …, *K*)^41^. We use a ‘hat’ to denote parameters which have been estimated through minimization of the χ^2^ cost function above and for the estimated (co)variance of such parameters. In the WLS method one uses weights ${R}_{ij}={\overline{R}}_{ij}={\delta }_{i,j}/{\overline{C}}_{ij}$, where *δ*_*i*,*j*_ is the Kronecker delta, and the (unbiased) sample “covariance matrix of the mean” is defined as ${\overline{C}}_{ij}={\overline{Q}}_{ij}/M$, with $\overline{{\boldsymbol{Q}}}$ being the sample covariance matrix

3 $${\overline{Q}}_{ij}=\frac{1}{M-1}\sum _{m=1}^{M}({y}_{i}^{(m)}-{\overline{y}}_{i})({y}_{j}^{(m)}-{\overline{y}}_{j})\mathrm{.}$$

While this specific choice of ***R*** is used in our applications, we note that the results in this section, including the new error formula below, is valid for arbitrary choices of ***R***. In Section A in Supplementary Information we elaborate on one “non-conventional” choice of ***R*** particularly adapted for BM.

The parameters, ${\hat{\theta }}_{a}$, obtained by minimizing *χ*^2^ in Equation (2), have a (co)variance ${{\rm{\Delta }}}_{ab}=\langle ({\hat{\theta }}_{a}-{\theta }_{a}^{\ast })({\hat{\theta }}_{b}-{\theta }_{b}^{\ast })\rangle$, where $\langle \ldots \rangle$ denotes ensemble average. Throughout this study we use a ‘star’ to denote exact parameter values, i.e., estimated values as *M* → ∞. The variances of the fitted parameter are ${\sigma }_{a}^{2}={{\rm{\Delta }}}_{aa}$. As noted in the Introduction, this covariance depends on the temporal correlations. For a stationary process, it is well-known how to estimate the variance of a mean in the presence of temporal correlations, typically by expressing the variance in terms of the sum or integral of the auto-correlation function^42,43^. In the present context, such an estimation corresponds to fitting to a constant, *f*_*i*_(*t*) = *θ*_1_, and assuming all correlation functions only depend on time differences.

We here extend the above-mentioned results to non-stationary processes and arbitrary fit functions by deriving the analogous expression for ${\hat{{\rm{\Delta }}}}_{ab}$ by using the full multivariate probability density for the fluctuations around mean values. Briefly, the covariance for the estimated parameters is defined ${\hat{{\rm{\Delta }}}}_{ab}=\langle ({\hat{\theta }}_{a}-{\theta }_{a}^{\ast })({\hat{\theta }}_{b}-{\theta }_{b}^{\ast })\rangle$ where $\langle F(\overline{{\boldsymbol{y}}})\rangle =\int F(\overline{{\boldsymbol{y}}})\rho (\overline{{\boldsymbol{y}}};{{\boldsymbol{\theta }}}^{\ast })d{\overline{y}}_{1}d{\overline{y}}_{2}\,\cdots \,d{\overline{y}}_{N}$ denotes an average over the multivariate probability density, $\rho \,(\overline{{\boldsymbol{y}}};{{\boldsymbol{\theta }}}^{\ast })$. We note that the dependence of the estimated parameters $\hat{{\boldsymbol{\theta }}}$ on $\overline{{\boldsymbol{y}}}$ is implicitly determined by the minimization condition ∂*χ*^2^/∂*θ*_*a*_ = 0. Now, because all ${\overline{y}}_{i}$ are averages over *M* identically distributed random numbers, for large *M*, it immediately follows from the multivariate central limit theorem that the function *ρ* takes the Gaussian form: $\rho (\overline{{\boldsymbol{y}}};{{\boldsymbol{\theta }}}^{\ast })={Z}^{-1}\exp (\,-\,{(\overline{{\boldsymbol{y}}}-{{\boldsymbol{y}}}^{\ast })}^{T}{{\boldsymbol{C}}}^{\ast -1}(\overline{{\boldsymbol{y}}}-{{\boldsymbol{y}}}^{\ast })/2)$ with normalization constant $Z={(2\pi )}^{N\mathrm{/2}}\sqrt{{\rm{\det }}({{\boldsymbol{C}}}^{\ast })}$^44^. Two complications that occur in evaluating ${\hat{{\rm{\Delta }}}}_{ab}$ in closed-form are that the $\overline{{\boldsymbol{y}}}$-dependence of $\hat{{\boldsymbol{\theta }}}$ is implicit, and, in general, non-linear. Both of these challenges are solved by making a Taylor series expansion of ${\hat{\theta }}_{a}-{\theta }_{a}^{\ast }$ in terms of $\overline{{\boldsymbol{y}}}-{{\boldsymbol{y}}}^{\ast }$ and implicitly using the minimization condition. The full derivation is given in Section A in Supplementary Information. The final result is the following estimator:

4a $${\hat{{\rm{\Delta }}}}_{ab}=\frac{{\hat{\varphi }}_{ab}}{M},$$

4b $${\hat{\varphi }}_{ab}=4\sum _{c,d}\sum _{i,j}{({\hat{{\boldsymbol{h}}}}^{-1})}_{ac}{\frac{{\rm{\partial }}{f}_{i}({\boldsymbol{\theta }})}{{\rm{\partial }}{\theta }_{c}}|}_{{\boldsymbol{\theta }}=\hat{{\boldsymbol{\theta }}}}{({{\boldsymbol{R}}}^{T}\bar{{\boldsymbol{Q}}}{\boldsymbol{R}})}_{ij}{\frac{{\rm{\partial }}{f}_{j}({\boldsymbol{\theta }})}{{\rm{\partial }}{\theta }_{d}}|}_{{\boldsymbol{\theta }}=\hat{{\boldsymbol{\theta }}}}{({\hat{{\boldsymbol{h}}}}^{-1})}_{db},$$

and

4c $${\hat{h}}_{ab}=2\sum _{i,j}{\frac{{\partial }^{2}{f}_{i}({\boldsymbol{\theta }})}{\partial {\theta }_{a}\partial {\theta }_{b}}|}_{{\boldsymbol{\theta }}=\hat{{\boldsymbol{\theta }}}}{R}_{ij}{{\rm{\Lambda }}}_{j}+2\sum _{i,j}{\frac{\partial {f}_{i}({\boldsymbol{\theta }})}{\partial {\theta }_{a}}|}_{{\boldsymbol{\theta }}=\hat{{\boldsymbol{\theta }}}}{R}_{ij}{\frac{\partial {f}_{j}({\boldsymbol{\theta }})}{\partial {\theta }_{b}}|}_{{\boldsymbol{\theta }}=\hat{{\boldsymbol{\theta }}}},$$

where the indices *a*,*b* = 1, …, *K*. Equation (4) gives a mathematically rigorous expression (to lowest order in 1/*M*) for the covariance of the estimated parameters, and is our key result. It allows us to accurately estimate the covariance of any parameter fitted by minimizing the cost function in Equation (2). Notice that the correlations in fluctuations around mean values enter through the quantity $\overline{{\boldsymbol{Q}}}$, which is estimated using the usual sample estimate above. In practice, our general formula, Equation (4) is simple to implement and computationally fast.

The new error estimation formula, Equation (4), reduces to previously known results in specific limits. (i) Neglecting the off-diagonal elements of $\overline{{\boldsymbol{Q}}}$ above we recover the WLS-ECE error estimation formula^9^. (ii) By setting $\overline{{\boldsymbol{R}}}={\overline{{\boldsymbol{C}}}}^{-1}$ above we recover the covariance estimation formula for CCM^10,12^. (iii) For a stationary process one seeks to fit a constant, *f*_*i*_(*θ*_1_) = *θ*_1_, to data. For such a case, the minimization procedure (solving ∂*χ*^2^/∂*θ*_1_ = 0 with *R*_*ij*_ = (1/*σ*^2^)*δ*_*i*,*j*_, where *σ* is the time-independent variance) yields ${\hat{\theta }}_{1}=(1/N){\sum }_{i}{\overline{y}}_{i}$, i.e., the parameter estimate is the mean of the data. The error estimation Equation (4), then reduces to the usual result^42,43^ $\hat{{\rm{\Delta }}}=(1/M){\sum }_{i,j}{\overline{Q}}_{ij}/{N}^{2}$ used, for instance, in analyzing Monte Carlo and molecular dynamics simulations. (iv) For linear fit functions, *f*_*i*_(***θ***) = *θ*_1_*t*_*i*_, Equation (4) reduces to previously known expressions (equation 5.253 in van den Bos^10^).

### Validation procedure

We tested the different fitting procedures on simulation data for our four prototype systems (generated as described in Section D in Supplementary Information). Estimated parameters, ${\hat{\theta }}_{a}$, were compared to their known exact values ${\theta }_{a}^{\ast }$ (see Section C in Supplementary Information). For BM, the MSD behaves as 〈[***x***(*t*) − ***x***(0)]^2^〉 = *f*(*θ*,*t*) = *θ*_1_*t*. The corresponding expression for FBM and CTRW is $\langle {[{\boldsymbol{x}}(t)-{\boldsymbol{x}}(0)]}^{2}\rangle =f({\boldsymbol{\theta }},t)={\theta }_{1}{t}^{{\theta }_{2}}$. For DHO (at critical damping and with the initial conditions *x*(0) = *x*_0_ and *v*(0) = 0), the mean position has the form 〈*x*(*t*)〉 = *f*(*θ*,*t*) = *x*_0_(1 + *θ*_1_*t*)exp(−*θ*_1_*t*).

For validating the WLS-ICE estimator for Δ_*ab*_, we generated *S* simulation sets (with *S* = 500) each consisting of *M* trajectories. Using these *S* × *M* trajectories, we obtained *S* number of parameter estimates ${\hat{\theta }}_{a}$. From these *S* estimates we calculate the covariance Δ_*ab*_ (using sample estimators), which then serves as true Δ_*ab*_ (“ground truth”). This true Δ_*ab*_ is then compared to estimates based on the WLS-ICE error formula, Equation (4) (which requires only one set of simulations), and the corresponding error estimates for WLS and CCM.

### Code availability

Computer codes (Python, Octave/MATLAB, and Lisp) which performs the associated fitting (determining ${\hat{\theta }}_{a}$) and error estimation (calculating ${\hat{{\rm{\Delta }}}}_{ab}$), using a set of measured observables for different trajectories and at different times as input, is freely available under the gnu General Public License (gpl)^45^ at http://cbbp.thep.lu.se/activities/wlsice/.

## Results

**Figure 1:**
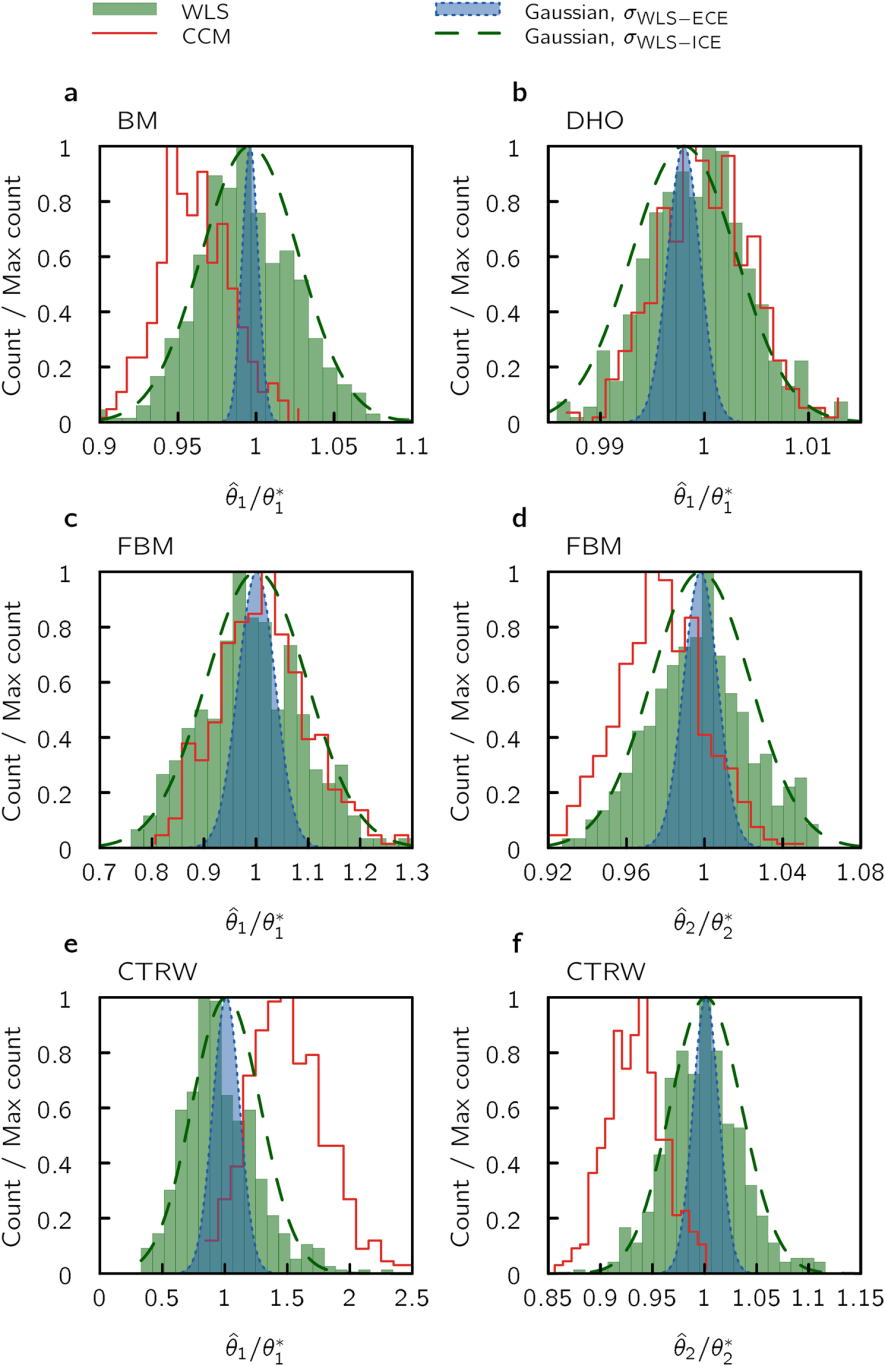
Histograms of fitted parameters for two WLS methods and CCM compared to theoretical predictions. Each method is tested on: (**a**) Brownian motion (BM), (**b**) damped harmonic oscillation (DHO) (**c**,**d**) fractional Brownian motion (FBM), and (**e**,**f**) continuous time random walk (CTRW). In each test, we generate *S* = 500 data sets, each consisting of *M* = 1000 trajectories sampled at *N* = 75 time points (histograms). Panel (a) shows the MSD prefactor (proportional to the diffusion constant) for BM, panel (b) shows DHO fitting parameter *θ*_1_, while panels (c–f) left and right panels show the MSD prefactor *θ*_1_, and the exponent *θ*_2_, respectively. For comparing WLS-ICE and WLS-ECE to the histograms based on the *S* data sets, we place Gaussian functions with their center positions at the mean of the WLS-fitted parameters. The widths of the Gaussians correspond to the parameter uncertainty estimated by the fit method (averaged over the *S* number of fits). The CCM fits for BM and CTRW exhibits a strong bias in the parameter value (not centered on the analytical prediction), and the WLS-ECE fit gives an error estimation, see Section B in Supplementary Information, that is much too small. The new WLS-ICE procedure (Methods) works well, i.e., exhibits negligible bias for all model systems and yields correct error estimation, Equation (4). The rather large number of trajectories (*M* = 1000) was used in order to avoid ill-conditioness and major bias issues for the CCM fitting, compare to Fig. 3. Results for a smaller ensemble size are found in Figure S2 in Supplementary Information, where we see that also for FBM there can be pronounced bias effects for CCM fitting. For simulation parameters, see Section D.5 in Supplementary Information.

**Figure 2:**
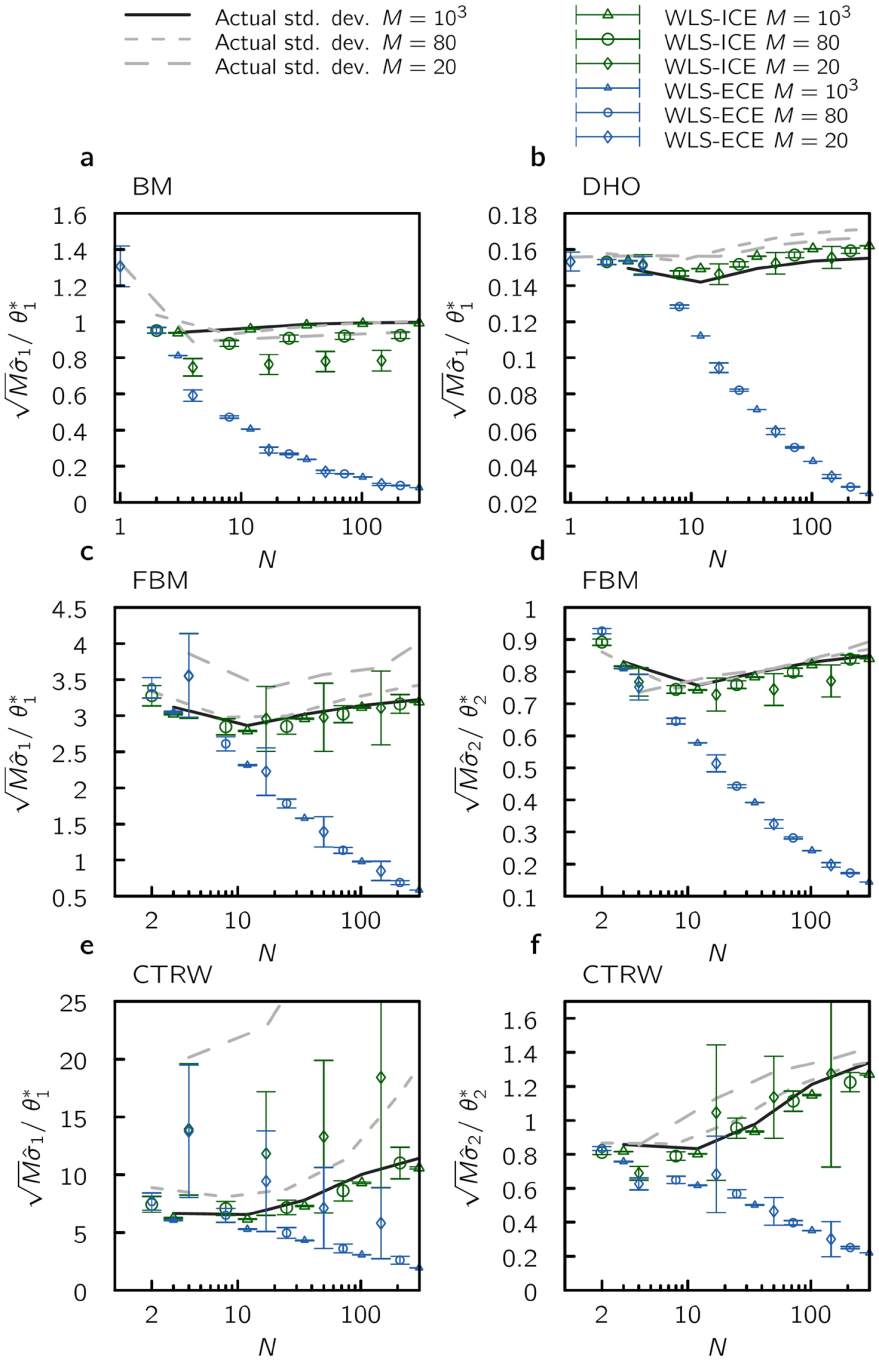
Error estimation. Standard deviation from the WLS-ECE and WLS-ICE parameter fits as a function of the number of sampling points, *N*, used in the fitting procedure (log-scale on the horizontal axis for visibility). Each method is applied to *S* = 500 realizations of data from (**a**) Brownian motion (BM), (**b**) damped harmonic oscillation (DHO), (**c**,**d**) fractional Brownian motion (FBM), and (**e**,**f**) continuous time random walk (CTRW). In conjunction we show the true standard deviation of each of these methods computed from the parameters from the fit (lines), i.e., the width seen in Fig. 1, but for an extended range of *N*. It is evident that the standard deviation from the WLS-ECE fit is far too small for almost all *N*. Error bars show standard error of the mean. For panels a-d there are small biases for *M* = 20 and *M* = 80 in the observable $\hat{\sigma }$, as compared to actual standard deviation. These biases can be removed using the jackknife procedure applied to Equation (4b), see Section G in Supplementary Information. For panel e, *M* = 20, there is discrepancy between the WLS-ICE estimate $\hat{\sigma }$, and the actual standard deviation; we assign this to slow convergence towards the asymptotic form for the multivariate distribution *ρ* (see Methods) for CTRW (see also Figure S2 in Supplementary Information). For simulation parameters, see Section D.5 in Supplementary Information.

Our first test of the fitting methods involve comparing histograms of fitted parameters for our four prototype systems (the number of trajectories, *M*, and number of sampling times, *N*, were kept fixed). For both CCM and WLS the *S* fitted values of a given parameter were binned to a histogram, see Fig. 1, and compared to a Gaussian centered on the mean of the estimated parameters with a variance from the average of the error estimates, using either the WLS-ECE or WLS-ICE method. For WLS, the histogram of fitted parameters is centered close to the true value (see also Figure S3 in Supplementary Information). However, only the WLS-ICE method gives a correct error estimation, Equation (4), as the predicted width of the WLS-ECE method, see Section B in Supplementary Information, is much too narrow. Clearly, the new error estimation of the WLS-ICE method performs extremely well. By contrast, the WLS-ECE method does not provide correct errors of the estimated parameters; this result extends beyond the chosen parameters for (*N*, *M*) in Fig. 1, and holds true under rather general conditions, see Fig. 2 (the exception is the prefactor for CTRW for very small *M*). Notice that while the parameters from the WLS-ICE and WLS-ECE methods are centered on the analytical prediction, this is not true for parameters from the CCM method, which show a strong bias (Fig. 1) for BM, FBM and CTRW (not for DHO). Thus, the WLS-ICE is the only method which yields an acceptable bias and correct error estimation for all model systems. Note that for the ensemble size used in Figure S2 in Supplementary Information, the distribution of fitted parameters is well described by a Gaussian, see Section F in Supplementary Information for a discussion on this topic. For a smaller ensemble size there are deviation from a Gaussian distribution, see Figure S2 in Supplementary Information, in particular for the prefactor for CTRW. From Fig. 2 we notice that the variance in the estimated parameter does not approach zero as *N* → ∞. Hence, the only way to decrease the variance in estimated parameters further is to increase *M* (the WLS estimator is consistent with respect to *M*).

As we have seen (Fig. 1), the CCM method gives a pronounced bias in the parameter estimate for a specific choice of the number of sampling times *N* and trajectories *M* for BM, FBM and CTRW systems, but not for DHO. In order to understand the generality of these findings, we numerically quantified the bias for an extended range of (*N*, *M*) values, and find that the pronounced bias for BM, FBM and CTRW (and lack of bias for DHO) is rather general, see Figure S3 in Supplementary Information. In Section E in Supplementary Information we investigate the expected bias for the CCM method further by analytical means. Indeed, we find that the parameter estimate from CCM fitting is unbiased for DHO. Mathematically, this result follows from the fact that the observable (mean position) used for the fitting is a linear function of the noise (this is in contrast to BM, FBM and CTRW, where the squared displacements are used as relevant observables). For BM, our analytical calculation in Section E in Supplementary Information shows that for large *N* the bias for CCM fitting becomes $\langle \hat{\theta }\rangle ={\theta }^{\ast }+DG(N)/M$, where *G*(*N*) ≈ −8*N*/(ln*N* + *γ* + 2ln2) and *γ* ≈ 0.5772 is the Euler-Mascheroni constant. Thus, with increasing number of sampling points *N*, the bias increases as *N*/ln*N* (see Figure S3 in Supplementary Information). The bias for CCM appears also in the FBM and CTRW systems, as seen in Figs 1 and S3 in Supplementary Information. A similar calculation for the WLS parameter estimate, see Section E in Supplementary Information, yields only a minor, essentially *N*-independent, bias with *G*(*N*) = −4(1 − 1/*N*) for BM.

**Figure 3:**
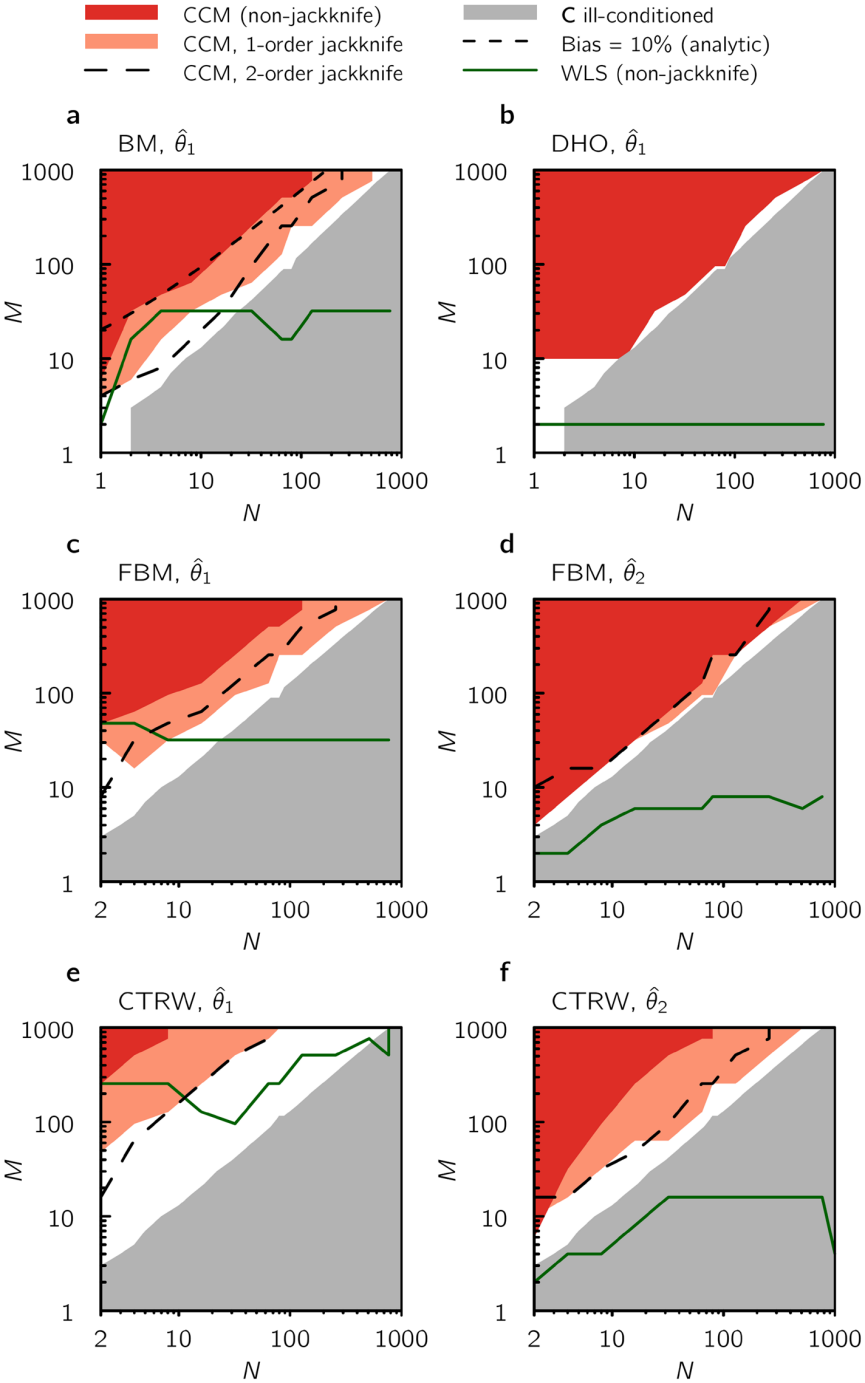
Phase space of reliable parameter estimation for CCM and WLS. For each of our example systems, (**a**) Brownian motion (BM), (**b**) damped harmonic oscillation (DHO) (**c**,**d**) fractional Brownian motion (FBM), and (**e**,**f**) continuous time random walk (CTRW), we investigate for which number of sampling times *N*, and number of trajectory realizations *M*, the fitting is more than 10% off from its analytical value, averaged over *S* = 500 simulations. As indicated, CCM is only reliable in a limited region (large *M*, small *N*), which can be extended by a first order jackknife correction. For BM we also include when the analytically predicted first order bias term for CCM, *G*(*N*), see Section E in Supplementary Information, gives a bias that is 10% of the true parameter value. We also show the boundary for when more than half of the *S* generated covariance matrices become ill-conditioned. Interestingly, for the CCM a second order jackknife generally does more harm than good compared to the first order, which we elaborate more on in Figure S4 in Supplementary Information. In contrast to CCM (non-jackknifed), the parameter estimations for the WLS method are acceptable for most *N*, *M* (region above the green curve), and can be extended even further using a jackknife approach (data not shown). For simulation parameters, see Section D.5 in Supplementary Information.

In order to further investigate practical implications of the pronounced bias for CCM fitting, as well as other known issues with the CCM method^14,15^, we quantified in what parts of phase space (*N*, *M*) the CCM fitting and WLS-ICE provides “acceptable” (see below) parameter estimation, see Fig. 3. First, we find that for large *N* and moderate to small *M*, the sample estimate for the covariance matrix ***C*** is ill-conditioned (the condition number is larger than the machine precision). In practice this means that it cannot be numerically inverted, as required in the CCM parameter estimation procedure, without uncontrollable numerical errors. Second, for parts of phase space where ill-conditioness is not an issue, we, rather generously, defined an acceptable fit as one where the bias is smaller than 10% (compared to the analytic value, ${\theta }_{a}^{\ast }$). We find that for BM, FBM and CTRW there is indeed a thin region of the (*N*, *M*)-phase space (large *M* and small *N*) where CCM works. For DHO, the bias effect is negligible, as previously noted. However, the ill-conditioness issue is as pronounced for DHO as for BM, FBM and CTRW. In contrast, for WLS ill-conditioness is not a problem (no matrix inversion is required in this procedure), and the bias in the parameter estimation is acceptable for most parts of the phase space. The bias inherent in the CCM method (for observables which are not linear functions of the noise (MSD for BM, FBM and CTRW)) can be reduced by applying the common jackknife procedure^46^, which removes bias terms proportional to 1/*M*, see Section G in Supplementary Information. By applying the (first-order) jackknife procedures to BM, FBM and CTRW (Fig. 3), we find that the bias is reduced which expands somewhat the region of the phase space where the CCM method may be used reliably. Note that the computational time is a factor *g* (i.e., the number of groups into which the trajectories are pooled) larger for the first-order jackknife procedure compared to the non-jackknife case. Finally, the jackknifing procedure can be extended to remove higher order bias terms (proportional to $\mathrm{1/}{M}^{n}$, with *n* = 2,3,…)^46^. However, for the present case there is no guarantee that these higher order terms have this functional form with respect to *M*, see Section E in Supplementary Information. Also, our results show that the second-order jackknife increased, rather than decreased, the bias in the parameter estimations for most parts of the phase spaces (Fig. 3). For BM, Figure S4 in Supplementary Information indicates that the reason for this is that the third order term (term proportional to 1/*M*^3^) is generally larger in amplitude (but of opposite sign) than the second order one. Higher order bias reduction comes at a computational price, since the number of numerical evaluations required for second order jackknife is *g*(*g* + 1)/2 times that of non-jackknifed parameter estimation. Due to these findings and the lack of a formal functional form for the bias, beyond the 1/*M* term (see above), we do not recommend applying the jackknife procedure beyond first order. Finally, we point out that the new error estimation formula, Equation (4), remains valid also for jackknifed parameters, see Section G in Supplementary Information.

In Figure S5 in Supplementary Information we investigated the “goodness of fit” for the WLS and CCM procedures using a standard *R*^2^ metric (see Section I in Supplementary Information). Examples of fitted curves are found in Figure S6 in Supplementary Information. A good fit is characterized by *R*^2^ ≈ 1. We find that, in this sense, the new method provides “good” fits. In contrast, the CCM method provides “bad” fits for BM, FBM and CTRW with ${R}^{2}\ll 1$ for large *N*. We point out that for the present type of data, *R*^2^ is only a heuristic goodness-of-fit metric — its distributional properties are not known for general fit functions and correlated data.

When computational times are not a concern, error estimation using bootstrap resampling (or the related jackknife error estimation procedure) are common method (see Section H in Supplementary Information)^47^. We here find that bootstrap resampling performs as well as WLS-ICE in general for our four models (jackknife error estimation is slightly worse), see Figure S7 in Supplementary Information. Thus, our numerical results indicate that for the type of observables and fit functions used in our model systems, the bootstrap can be used for calculating the variance for parameters estimated through *χ*^2^ minimization. However, we point out that such resampling techniques require us to repeat the *χ*^2^ minimization several (herein, 100) times (the WLS-ICE method requires only one *χ*^2^ minimization). Such minimization can be computationally costly, especially for the case when the number of unknown parameters is large. Moreover, one must bear in mind that the bootstrap method is in general a heuristic method (there are cases when it does not apply^47^).

As a final alternative to the WLS-ICE method, we now briefly turn to error estimation using subsampling^43^. Subsampling refers to the method of choosing sampling times sufficiently sparsely in order to make the data points essentially uncorrelated (the “brute force” method in Figure S1 in Supplementary Information is an extreme case of subsampling where only one data point per trajectory is kept). After subsampling, error analysis is performed using standard error analysis for independent data. In order to properly choose *N* within this method, *N* is systematically decreased until the variance saturates to a constant, which is assumed to be the true variance^43^. Notice for stationary time series, rather than reducing the number of sampling times, one can make full use of the data through the blocking method^42^. For non-stationary processes the blocking method cannot be used, however. Figure 2 shows how estimated errors from our WLS-ECE and WLS-ICE analyses depend on the number of data points used, *N*. We find that temporal correlations are so strong that the WLS-ECE method underestimates the errors down to very small *N*. Moreover, finding a sufficiently small *N* is difficult, since the error does not in general saturate to a constant level as *N* is reduced. These problems are circumvented by instead using the error estimation from the WLS-ICE method (i.e., using Equation (4) instead of the WLS-ECE equations in Section B in Supplementary Information).

As a final test of our method, we now turn to “real world” data. To that end, we use particle tracking data used in a competition for testing particle tracking software where 14 teams world-wide participated^48^. We choose to analyze this data set for two reasons. First, it served as standard benchmark data within the particle tracking community. Second, since these movies are based on noisified and pixelated simulations (aiming to mimic actual experimental data), we know the values of the underlying model parameters. We used their Supplementary Videos 1 (medium particle density), 5 (low particle density) and 6 (high particle density). All these movies correspond to BM of vesicles for which the expected MSD for the data sets are 〈[***x***(*t*) − ***x***(0)]^2^〉 = *f*_B*M*_(*θ*,*t*) = *θ*_1_*t*, with *θ*_1_ = 2*dD* = 8. For particle detection in the movies and linking of particle positions into trajectories we used Method 1^48^, i.e., the tracking method described by Sbalzarini *et al*.^49^, and implemented as the ImageJ plugin “Particle Tracker” by the MOSAIC group^50^. Parameter settings for the plug-in are listed in Section J in Supplementary Information. For each video we extracted trajectories which were subsequently cut into trajectories consisting of 7 discrete process times (there is no memory in BM, so the start time is inessential). Notice that for the higher particle density, fewer sufficiently long trajectories were produced as compared to the low density scenario (values for *M* are listed in Table 1). We subsequently divided the trajectories for each movie into two data sets each with *M* trajectories. For the fitting procedures the first process time point, *t*_0_ = 0, was discarded (since at *t*_0_ the position is precisely known, the variance = 0 and can not be used as a weight in Equation (2)), thus leaving us with *N* = 6 sampling times. Results for the estimated parameters, ${\hat{\theta }}_{1}$ and associated standard deviation, $\hat{\sigma }$ are found in Table 1. We notice that the CCM method fails at predicting the correct parameter for high and medium particle densities. This finding is simply due to the smaller ensemble size for these cases which, in turn, is a result of the tracking software’s inability to track and link particles in high and medium density settings. Comparing the WLS-ECE and WLS-ICE method, we see that the WLS-ECE underestimates the error by factors ≈ 2 for all movies. While, this underestimation may seem minor it will affect conclusions drawn from particle tracking data (see discussion in Introduction), in particular it is noteworthy that for the WLS-ECE method only 2 out of 6 estimates fall within 2*σ* (confidence level 95%) of the expected result (=8). In contrast, for the WLS-ICE all six observed parameter estimations for *θ*_1_ fall within 2*σ* of the expected value.

**Table 1:** Results of the three fitting methods for “real world” particle tracking data.

Description Video Number of trajectories		Low density S5 *M* = 310		Medium density S1 *M* = 16		High density S6 *M* = 5	
**Method**	Observable						
WLS-ICE	${\hat{\theta }}_{1}$	8.49	8.63	11.41	8.14	7.60	5.45
	$\hat{\sigma }$	0.38	0.38	2.17	1.81	2.53	1.93
WLS-ECE	${\hat{\theta }}_{1}$	8.49	8.63	11.41	8.14	7.60	5.45
	$\hat{\sigma }$	0.20	0.19	1.25	0.93	1.56	1.00
CCM	${\hat{\theta }}_{1}$	8.63	8.33	10.83	3.79	ill-cond.	ill-cond.
	$\hat{\sigma }$	0.37	0.35	1.14	1.60	ill-cond.	ill-cond.

Particle trajectories where extracted from the “Vesicle” Supplementary videos from the article by Chenouard *et al*.^48^ using the “Particle Tracker” software (MOSAIC group). The trajectories where cut into shorter trajectories, all of length 7 discrete process times. The short trajectories were then divided into two independent sets of size *M*. We then performed fitting using the WLS-ICE, WLS-ECE and CCM methods for BM, discarding the first process time point, resulting in *N* = 6 sampling times. Expected parameter value is *θ*_1_ = 8 (data are noisified and pixelized simulations with known properties). Since *M* was very small for video S6, we applied the jackknife procedure both in parameter and error estimation (all videos). Results before jackknifing are found in Table S1 in Supplementary Information. We notice that the CCM method gives ill-conditioness issues for the high density movie, where few trajectories could be extracted. The WLS-ECE method underestimates the error as compared to WLS-ICE method.

Let us finally briefly discuss how well one is expected to be able to estimate a parameter based on experimental/simulation data. For model matching procedures (see Introduction), the Cramer-Rao bound is useful by providing an expression for the smallest possible variance in the estimated parameter^10^. For the case of BM, optimal estimators (i.e., estimators which reach the Cramer-Rao bound) based on the measured displacements have been derived for model matching type fitting^19–21^. For function fitting, the question is rather whether an optimal cost function, i.e., an optimal weight matrix ***R***, can be found (see Equation (2)). If the covariance matrix for the process is independent of the inferred parameters (up to a proportionality constant), and for linear fit functions, then the generalized least squares method can be shown to be optimal among unbiased WLS methods^51^. Since the generalized least squares method requires as input the inverse of the true covariance matrix, it can be viewed as a hybrid method in between model matching and function fitting. In Figure S8 in Supplementary Information we show results for the generalized least squares for BM (we use the term BMALS – Brownian motion adapted least squares) where we see that, indeed, the variance in estimated parameter value is smaller for BMALS as compared to WLS-ICE, although the difference is not dramatic. Also notice that for *M* and *N* values where the CCM “works” (acceptable bias, see Fig. 3) the variance in estimated parameters for CCM and BMALS agree, as it should.

## Discussion, Conclusion and Outlook

A common task in many fields of science is that of fitting a model to the time-evolving mean of some observable. Since fluctuations around observed mean values, calculated based on trajectories, are in general correlated in time, the error estimates provided by a “standard” weighted least squares (WLS-ECE) fit can be more than one order of magnitude too small, see Fig. 2. Further, the correlated chi-square method (CCM), involving numerical inversion of a noisy covariance matrix, often show numerical instabilities (ill-conditioning) or a strong bias in the fitted parameters, see Fig. 3. To overcome these problems, we derived a new error estimation formula, see Equation (4), for weighted least squares fitting, which does not require inversion of a noisy covariance matrix. With this formula at hand, a simple, yet accurate, function fitting procedure, WLS-ICE, can be followed: (A) perform a weighted least squares fit to the data, (B) use the new formula to estimate the errors. We demonstrated on four simulated prototype systems that the WLS-ICE method provides robust results, with a negligible bias in the fitted parameters and accurate error estimates. Our method’s estimated errors are comparable to errors estimated using bootstrap and jack-knife resampling for the four model systems. A strength of our method is that the fitting procedure does not have to be repeated multiple times.

We separated between two types of parameter estimation procedures: model matching where a full stochastic model is matched to the data, and function fitting in which a full stochastic model is not known and one rather seeks to fit a function to the chosen ensemble-averaged observables. The weighted least-squares method is a procedure of function fitting type.

We have in this study not discussed methods for dealing with experimental errors, such as missing data etc. Such errors depend on the experimental setup and typically have to be dealt with in different ways depending on setup. For the single-particle tracking field (one of the application fields of our results), two major sources of experimental errors are: effects due to the finite size of pixels in cameras used to record the trajectory and motional blur effects (in a single time frame, a fluorescent molecule moves while being imaged). Methods for correcting these types of errors are discussed by Savin *et al*.^52^, Martin *et al*.^53^, Berglund^19^ and Calderon^54^.

Parameter estimation through *χ*^2^ minimization is ubiquitous throughout many fields of science, and we hope that our method and publically available software will be found useful in these fields.

## Electronic supplementary material

Supplementary Information
