# Supplementary material for: Fitting a function to time-dependent ensemble averaged data
Source: Sci Rep. 2018 May 3;8:6984. doi: 10.1038/s41598-018-24983-y (PMC5934400; doi:10.1038/s41598-018-24983-y)
Supplement: Supplementary file 1 — Supplementary Information [file 41598_2018_24983_MOESM1_ESM.pdf]

Supplementary Information: Fitting a function to time-dependent ensemble averaged data

Karl Fogelmark[1], Michael A. Lomholt[2], Anders Irbäck[1], Tobias Ambjörnsson[1,*]
[1] Computational Biology and Biological Physics, Department of Astronomy and Theoretical Physics, Lund University, 223 62 Lund, Sweden
[2] Department of Physics, Chemistry and Pharmacy, University of Southern Denmark, Campusvej 55, 5230 Odense M, Denmark
* tobias.ambjornsson@thep.lu.se

Supplementary Figures

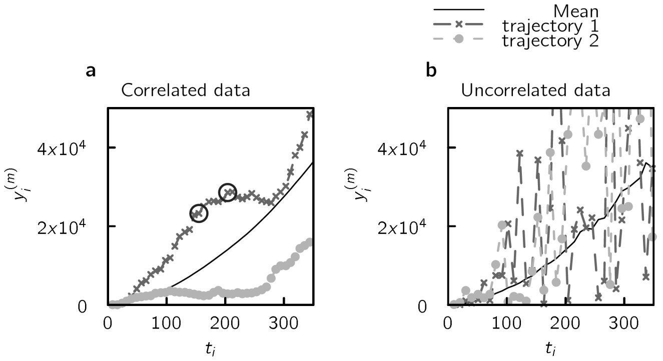

Supplementary Figure S1. Correlations in fluctuations around ensemble averages for real trajectories compared to uncorrelated fluctuations (synthetic data). The displacement squared $y_i^{(m)} = [\boldsymbol{x}^{(m)}(t_i) - \boldsymbol{x}^{(m)}(0)]^2$ for fractional Brownian motion (FBM) as a function of time, t, for two trajectories, labeled by m, and the mean of a large ensemble ($M = 10^3$) of trajectories. Panel (**a**) shows actual trajectories which exhibit strong temporal correlation, meaning: if we are above the mean for some time point on a trajectory, we are likely to still be above the mean for time points close to it (circled). In panel (**b**) we have constructed “synthetic” trajectories for comparison by only using one data point from each real trajectory, and "throw away" the rest, resulting in (computationally expensive) uncorrelated data. That is, within this “brute force” method, to generate a single uncorrelated trajectory of N sampling points, we need to use the same amount of real trajectories, and throw away all data points save one. Data was generated from a one-dimensional FBM simulation with Hurst parameter $H = 0.9$, see Supplementary Methods section C.3.

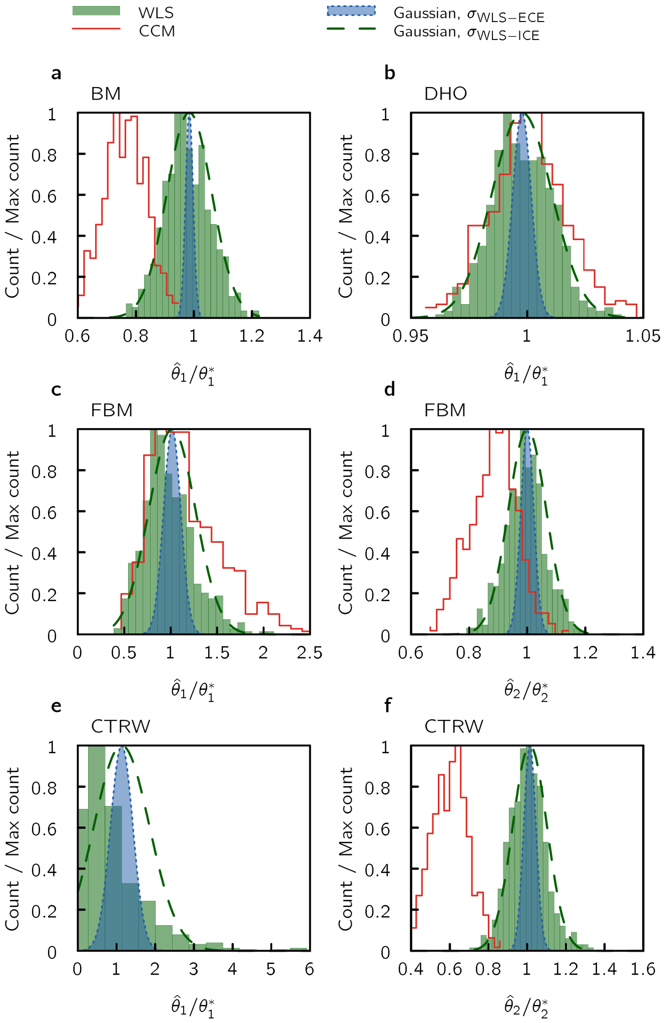

Supplementary Figure S2. Histograms of fitted parameters for two WLS methods and CCM compared to theoretical predictions for a small ensemble size. All panels are identical to those in Figure 1 in the main text except that we here only used $M = 150$ trajectories (instead of $M = 1000$). In panel **e** (the CTRW prefactor), the CCM fitting procedure gave a vastly incorrect parameter estimate ($\langle\overline{\theta}_1\rangle/\theta_1^* = 13.2$) and the associated histogram is therefore not displayed. Due to the smaller M value used here as compared to Figure 1 in the main text the histogram of fitted parameters are non-Gaussian for panel **e**, see Supplementary Methods Sec. F for a discussion on this topic. The other panels converged to normal distributions for smaller M values. Examples of parameter fits to the MSD data are shown in Fig. S6.

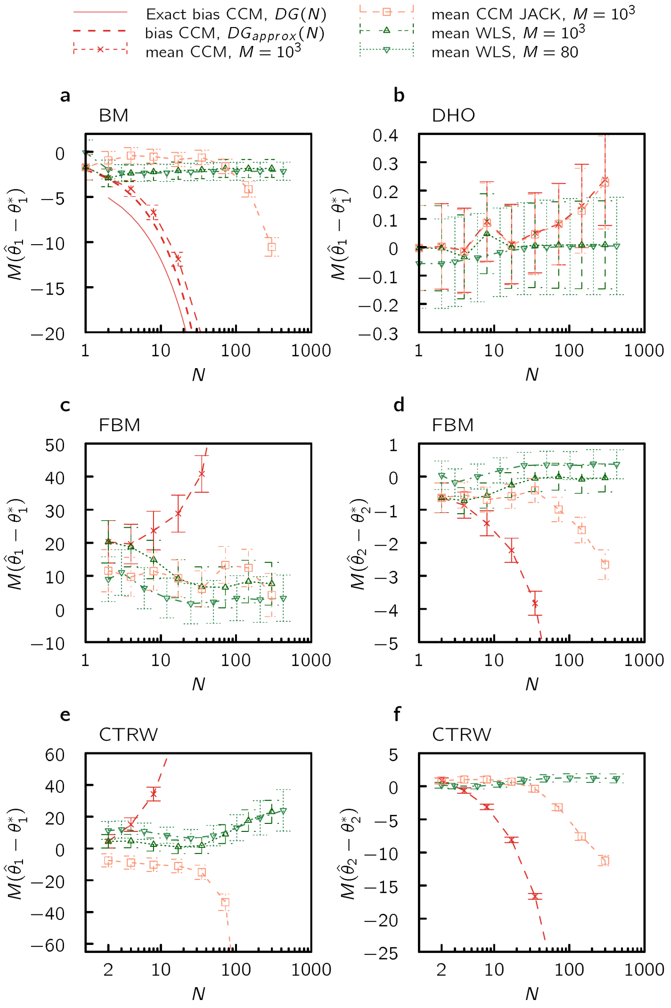

Supplementary Figure S3. Bias in the parameter fit. The residual bias in the fit (multiplied by the number of trajectories M) as a function of sampling points, N (log-scale for the horizontal axis for visibility), averaged over parameters from fitting to $S = 500$ realizations of the mean. (**a**) For the Brownian motion (BM) CCM fit, the analytical prediction, $G(N)$, (full line) for the first order bias follows the observed bias for $M = 10^3$, data (Supplementary Methods section E.3). For (**b**) damped harmonic oscillation (DHO) the bias in CCM and WLS are both small, but for (**c**–**d**) fractional Brownian motion (FBM), and (**e**–**f**) continuous time random walk (CTRW), the bias term in CCM is much larger than the WLS bias. The bias can be alleviated to some degree by a Jackknife procedure. Error bars show standard error of the mean. For simulation parameters, see Supplementary Methods section D.5.

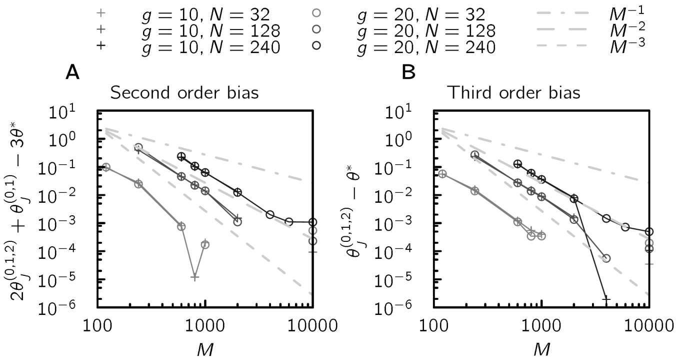

Supplementary Figure S4. High order bias contribution in CCM fitting for BM. The bias in the parameter estimation is commonly assumed to be of the form $\hat{\theta} = \theta^* + a/M + b/M^2 + c/M^3 + \mathscr{O}(M^{-4})$, see Supplementary Methods section E. In panel (**a**) the vertical axis shows the (negative) second order bias term $-b/M^2$, and in (**b**) the (positive) third order term, c/M^3, for three different number of sampling times N. Note that these are of comparable magnitude, but opposite sign. Thus a second order jackknife, which removes terms proportional to a/M and b/M^2, may yield more unfavorable results than a first order jackknife, which only removes the a/M term. We note that the slope of the second order bias term approximately corresponds to M^{-2}, and the third order is slightly more. For panel (a) the second order bias was extracted combining equation (S122) and equation (S124), to give $-b/M^2 = 2\theta_J^{(0,1,2)} + \theta_J^{(0,1)} - 3\theta^*$, and for panel (b) we have $(\theta_J^{(0,1,2)} - \theta^*) = c/M^3$, which follows immediately from equation (S124). For simulation parameters, see Supplementary Methods section D.5.

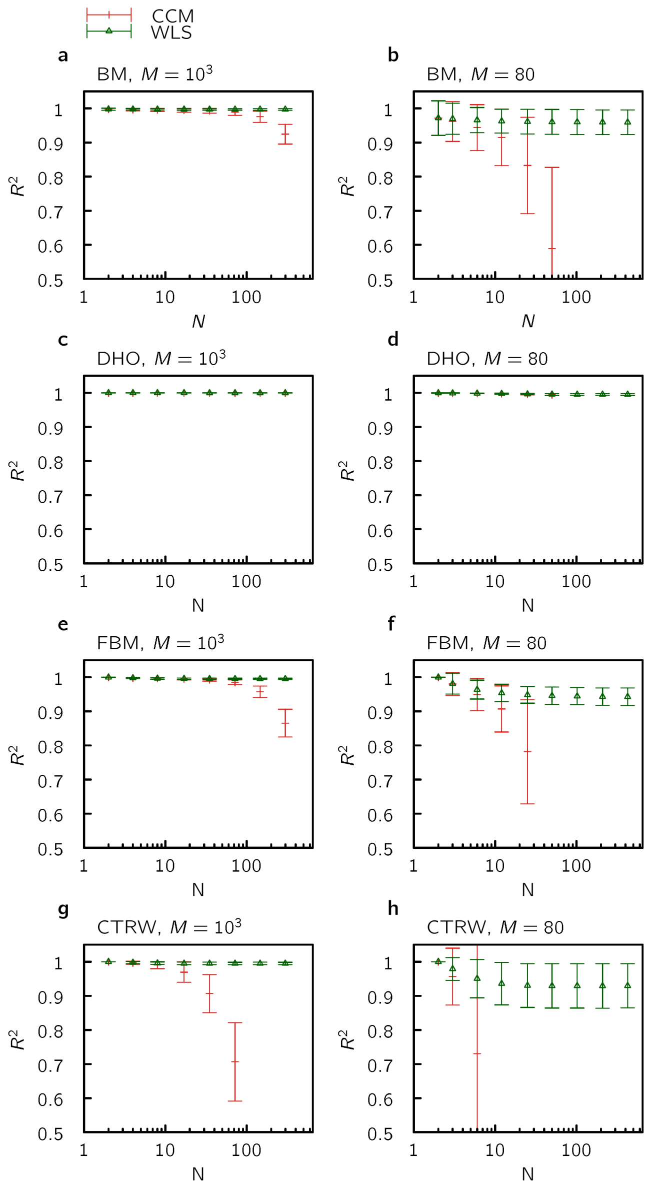

Supplementary Figure S5. Heuristic goodness-of-fit using the the coefficient of determination, R^2. The quality of the CCM and WLS fits are heuristically quantified by the coefficient of determination, R^2, as a function of sampling points, N (horizontal axis on log-scale for visibility), for our four prototype systems: (**a**–**b**) Brownian motion (BM), (**c**–**d**) damped harmonic oscillation (DHO), (**e**–**f**) fractional Brownian motion (FBM), and (**g**–**h**) continuous time random walk (CTRW). A perfect fit yields unit value, while a bad fit results in $R^2 \ll 1$ (see Supplementary Methods, Sec. I). The number of trajectories used in the ensemble average was either $M = 10^3$ (left), or $M = 80$ (right). All data was averaged over $S = 500$ realizations, with standard deviation given by the error bars. For panels (**b**,**f**) only a few data points could be obtained, due to numerical instability of CCM, and for panels (**g**,**h**) $R^2 < 0$ for larger N. For simulation parameters, see Supplementary Methods section D.5.

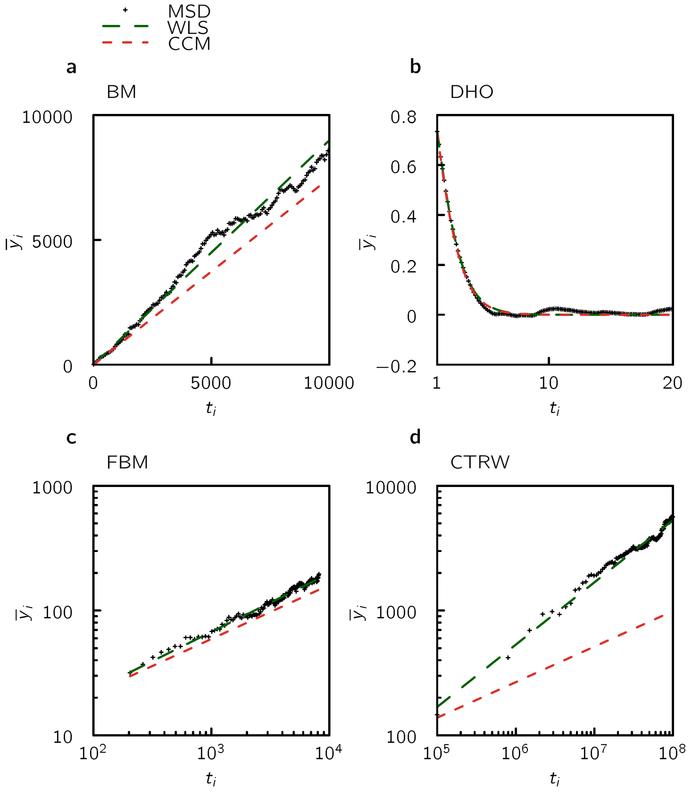

Supplementary Figure S6. Example of a fit to the mean of ensemble data for the WLS and CCM methods. An illustrative example of a typical fit to average ensemble trajectory data, based on $M = 150$ trajectories, for (**a**) Brownian motion (BM), (**b**) damped harmonic oscillation (DHO), (**c**) fractional Brownian motion (FBM), and (**d**) continuous time random walk (CTRW). The model parameters were fitted to the data using either WLS or CCM fitting procedure, for $N = 75$, $M = 150$. For CCM fitting to the FBM data, we see that although the exponent is almost the same, the pre-factor is inaccurate. For CCM fitting to CTRW data, both exponent and pre-factor is poor. For simulation parameters, see Supplementary Methods section D.5.

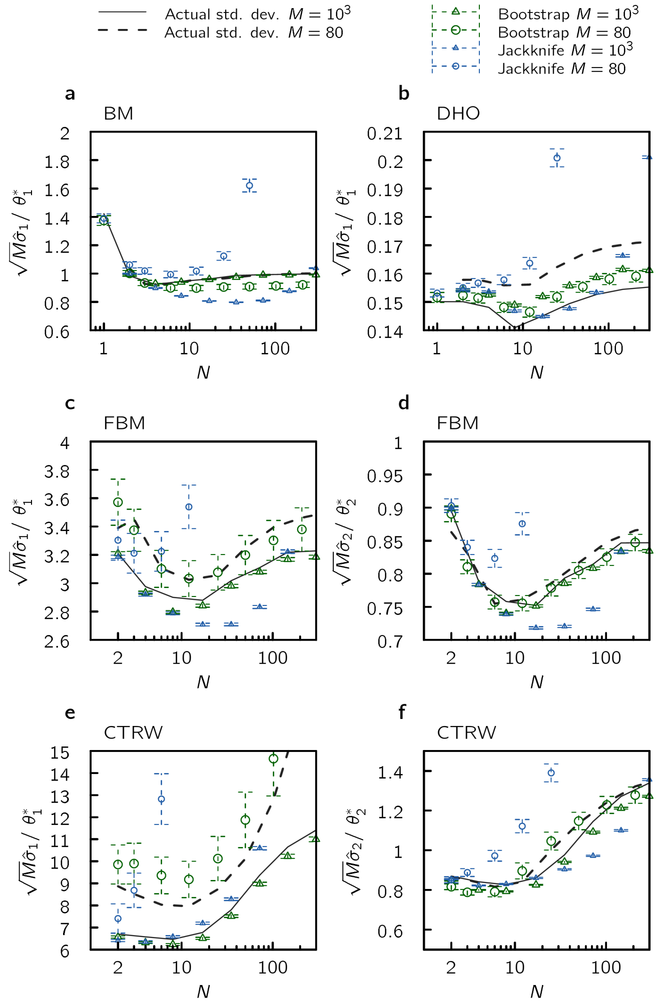

Supplementary Figure S7. Error estimation using bootstrap resampling and jackknife error estimation. Standard deviation for the parameter fits as a function of the number of sampling points, N, used in the fitting procedure. Each method is applied to $S = 500$ realizations of data from (**a**) Brownian motion (BM), (**b**) damped harmonic oscillation (DHO), (**c–d**) fractional Brownian motion (FBM), and (**e–f**) continuous time random walk (CTRW). The associated standard deviation in parameter estimates serve as "actual" standard deviation. These actual values are compared to estimates using bootstrap resampling and jackknife error estimation procedures, see Supplementary Methods, Sec. H. We see that the bootstrap method gives rather reliable error estimates which are similar to that of the WLS-ICE procedure, compare to Figure 2 in the main text. However, note that the bootstrap method is associated with a substantially larger computational time compared to the WLS-ICE. The jackknife error estimation performs worse than bootstrap resampling in general. For the jackknife error estimation, we used 100 groups. For the bootstrap results, trajectories were resampled with replacement and the χ^2 minimization performed 100 times. For simulation parameters, see Supplementary Methods section D.5.

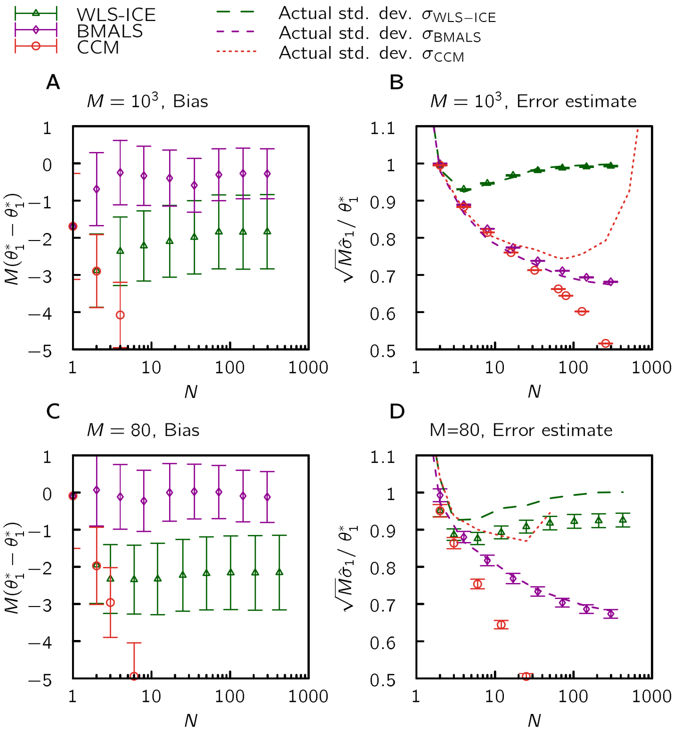

Supplementary Figure S8. Bias and variance of Brownian motion adapted least squares (BMALS) compared to the WLS-ICE and CCM methods. We show the bias in parameter fit (left panels) and their variance compared to estimates from fitting procedure (right panels), as a function of the number of sampling times, N. The MSD based on two different data sizes, M (number of trajectories) was considered: (**a**,**b**) $M = 10^3$ and (**c**,**d**) $M = 80$; averaged over $S = 500$ realizations. Notice the lower variance in BMALS as compared to WLS-ICE, and that as M is increased the CCM variance approach the variance for BMALS. The BMALS is a hybrid between model matching and function fitting procedures as it requires the true covariance matrix as input. Error bars show standard errors of the mean. For simulation parameters, see Supplementary Methods section D.5.

Supplementary Tables

**Description**		Low density		Medium density		High density	
**Video**		S5		S1		S6	
**Number of trajectories**		$M = 310$		$M = 16$		$M = 5$	
**Method**	Observable						
WLS-ICE	$\hat{\theta}_1$	8.46	8.62	10.30	7.70	6.75	5.22
	$\hat{\sigma}$	0.38	0.38	1.88	1.64	2.56	1.93
WLS-ECE	$\hat{\theta}_1$	8.46	8.62	10.30	7.70	6.75	5.22
	$\hat{\sigma}$	0.20	0.19	1.14	0.86	1.61	1.04
CCM	$\hat{\theta}_1$	8.53	8.27	9.25	3.84	ill-cond.	ill-cond.
	$\hat{\sigma}$	0.36	0.35	1.01	1.17	ill-cond.	ill-cond.

Supplementary Table S1. Results of the three fitting method for "real world" particle tracking data, without jackknife. Results shown are for the same data as in Table 1 in the main text, but here before the jackknife procedures were applied. Comparing to Table 1 in the main text we see that biases is rather large for video S6 (few trajectories) but minor for video S5 (large number of trajectories).

**Abbreviation**		**Comment**
WLS-ICE	weighted least squares including correlations in error estimation	new method
WLS-ECE	weighted least squares excluding correlations in error estimation	old method
CCM	correlated chi-square method	old method
BM	Brownian motion	zero-mean process without memory
DHO	damped harmonic oscillation	process with a time-dependent mean
FBM	fractional Brownian motion	zero-mean process with memory
CTRW	continuous time random walk	zero-mean, ageing process

Supplementary Table S2. List of abbreviations.

Contents

A Weighted Least Squares Including Correlations in Error estimation (WLS-ICE) 11
A.1 Parameter estimation . . . 11
A.2 Error estimation . . . 12
B Review of previous fitting procedures 12
B.1 WLS-ECE fitting . . . 13
General fit functions • Linear fit functions
B.2 CCM fitting . . . 14
General fit functions • Linear fit functions
C Prototypical model systems 15
C.1 Brownian motion . . . 15
C.2 Damped Harmonic Oscillation in a heat bath (DHO) . . . 16
C.3 Fractional Brownian motion . . . 18
C.4 Continuous time random walk (CTRW) . . . 18
D Simulation procedures 19
D.1 Brownian motion (BM) . . . 19
D.2 Damped harmonic oscillation (DHO) . . . 19
D.3 Fractional Brownian motion (FBM) . . . 19
D.4 Continuous time random walk (CTRW) . . . 19
D.5 Simulation parameters . . . 19
E Bias effects in parameter estimation 20
E.1 The origin of bias . . . 20
E.2 Bias in parameter estimation of CCM for linear fit functions . . . 20
E.3 Bias in parameter estimation of CCM for BM . . . 21
Asymptotic expansion
E.4 Bias in parameter estimation of WLS for BM . . . 23
E.5 Lack of bias for BMALS . . . 24
E.6 Lack of bias in parameter estimation of CCM for DHO . . . 24
F Approximate distribution for the estimated parameters 25
G Jackknife bias reduction 25
G.1 First order jackknife bias reduction . . . 26
G.2 Second order jackknife bias reduction . . . 26
G.3 Variance for jackknife-bias-reduced estimators . . . 26
First order jackknife bias reduction • Second order jackknife bias reduction
H Estimation of errors on estimated parameters, using jackknife and bootstrap procedures 28
H.1 Jackknife error estimation . . . 28
H.2 Bootstrap error estimation . . . 28
I Coefficient of determination 28
J Settings in "Particle Tracker" plug-in 29
References 29

Supplementary Methods

In this Supplementary Methods, details of the derivations, simulations and methods are provided. For convenience, Table S2 lists all abbreviations used herein.

A Weighted Least Squares Including Correlations in Error estimation (WLS-ICE)

We here describe our new fitting procedure, the WLS-ICE method, in detail. As demonstrated in the main text, the previous standard methods for fitting of ensemble averages, the WLS-ECE or CCM procedures (section B), are of limited general applicability for fitting of correlated data: the WLS-ECE method assumes data points are independent resulting in flawed error estimation, whereas the CCM method (involving inversion of a noisy sample covariance matrix) provides ill-conditioned results or strong bias in the parameter estimation. We here formulate the problem at hand as a minimization of a "cost function", χ^2, which can be chosen rather general. Minimizing this cost function provides an estimate, $\hat{\boldsymbol{\theta}}$, for the model parameters of interest. However, unlike the WLS-ECE fitting procedure, where fluctuations around mean values are assumed to be independent, we use the full multivariate probability density function for the mean values, eq. (S8) (which is Gaussian due to the multivariate central limit theorem), when estimating the standard error and covariance in the fitted parameters. This provides a mathematically rigorous way of avoiding the problems with previous fitting methods.

A.1 Parameter estimation

The cost function used herein is a χ^2 functional (eq. (2) in the main text) on the form:

$$\chi^2 = (\boldsymbol{f} - \bar{\boldsymbol{y}})^T \boldsymbol{R}\,(\boldsymbol{f} - \bar{\boldsymbol{y}}), \tag{S1}$$

where $\bar{\boldsymbol{y}} = (\bar{y}_1, \dots, \bar{y}_N)$, $\boldsymbol{f} = (f_1, \dots, f_N)$, $f_i = f(T_i; \boldsymbol{\theta})$ with sampling times T_i $(i = 1, \dots, N)$ and where $(\dots)^T$ denote transpose. We find the best parameters $\hat{\boldsymbol{\theta}}$ by minimizing χ^2, i.e., by solving:

$$\left.\frac{\partial \chi^2}{\partial \theta_a}\right|_{\boldsymbol{\theta}=\hat{\boldsymbol{\theta}}} = 0 = 2\sum_{i,j} \left.\frac{\partial f_i(\boldsymbol{\theta})}{\partial \theta_a}\right|_{\boldsymbol{\theta}=\hat{\boldsymbol{\theta}}} R_{ij}(f_j(\hat{\boldsymbol{\theta}}) - \bar{y}_j), \tag{S2}$$

where $a = 1, \dots, K$. As in the main text, a 'bar' denotes a sample estimator, a 'hat' denotes parameters obtained through χ^2 minimization, and a 'star' is used to denote the true value of a parameter. For a linear fit function, $f_i(\theta_1) = \theta_1 T_i$, eq. (S2) can be solved analytically:

$$\hat{\theta}_1 = \frac{\bar{\boldsymbol{y}}^T \boldsymbol{R}\boldsymbol{T}}{\boldsymbol{T}^T \boldsymbol{R}\boldsymbol{T}}. \tag{S3}$$

Note that the positive definite symmetric matrix $\boldsymbol{R}$ in eq. (S1) could potentially be custom made for particular applications. In the main text the observables $\bar{y}_i$ are mean positions or mean square displacements at different sampling times, T_i. We note, however, that our WLS-ICE procedure is valid for any type of ensemble averaged observables (the matrices $\overline{\boldsymbol{C}}$ and $\overline{\boldsymbol{Q}}$ below are then the covariance matrix for those particular observables).

For the matrix $\boldsymbol{R}$, we consider three main choices:

1. Correlated Chi-Square Method (CCM): Here we make use of the full covariance matrix, (see section B.2):

$$\boldsymbol{R} = \overline{\boldsymbol{R}}^{[CCM]} = \overline{\boldsymbol{C}}^{-1}, \tag{S4}$$

where $\overline{\boldsymbol{C}}$ is the covariance matrix of the mean, $\overline{\boldsymbol{C}} = \overline{\boldsymbol{Q}}/M$, as defined in eq. (3) in the main text.

2. Weighted least squares (WLS): Here we only make use of the diagonal elements,

$$R_{ij} = \overline{R}_{ij}^{[WLS]} = \delta_{i,j}/\overline{C}_{ii}, \tag{S5}$$

where $\delta_{i,j}$ is the Kronecker delta-function.

3. Brownian motion adapted least squares (BMALS): Finally we probe our fitting method by the following choice:

$$\boldsymbol{R} = \overline{\boldsymbol{R}}^{[BMALS]} = \frac{1}{M}\boldsymbol{Q}_{\mathrm{BM}}^{*}{}^{-1}, \tag{S6}$$

where $\boldsymbol{Q}_{\mathrm{BM}}^{*}$ is the exact covariance matrix for BM, see eq. (S38). For comparison of the BMALS method to WLS-ICE, please see Supplementary Figure S8.

A.2 Error estimation

The covariance for the estimated parameters (i.e., the parameters $\hat{\boldsymbol{\theta}}$ obtained by solving eq. (S2)) is defined

$$\hat{\Delta}_{ab} = \langle (\hat{\theta}_a - \theta_a^*)(\hat{\theta}_b - \theta_b^*) \rangle, \tag{S7}$$

where $\langle F(\bar{\mathbf{y}}) \rangle = \int F(\bar{\mathbf{y}}) \rho(\bar{\mathbf{y}}; \boldsymbol{\theta}^*) d\bar{y}_1 d\bar{y}_2 \cdots d\bar{y}_N$ denotes an average over the multivariate probability density, $\rho(\bar{\mathbf{y}}; \boldsymbol{\theta}^*)$. Due to the multivariate central limit theorem (note that $\bar{\mathbf{y}}$ is a sum of M identically distributed random numbers), for large M this probability density is a multi-variate Gaussian:

$$\rho(\bar{\mathbf{y}}; \boldsymbol{\theta}^*) = Z^{-1} \exp\left(-\frac{1}{2} (\bar{\mathbf{y}} - \mathbf{y}^*)^T \mathbf{C}^{*-1} (\bar{\mathbf{y}} - \mathbf{y}^*) \right), \tag{S8}$$

with normalization constant $Z = (2\pi)^{N/2} \sqrt{\det(\mathbf{C}^*)}$[1] and $\mathbf{C}^* = \mathbf{Q}^*/M$, where $\mathbf{Q}^*$ is the exact covariance matrix.

In order to derive an explicit expression for $\hat{\Delta}_{ab}$ we follow the lines of thought of Gottlieb *et al.*[2] and make a first order Taylor series expansion of the estimated parameter values in terms of deviations of the estimated $\bar{\mathbf{y}}$ from their true values:

$$\hat{\theta}_a - \theta_a^* = \sum_k \left. \frac{\partial \hat{\theta}_a}{\partial \bar{y}_k} \right|_{\bar{\mathbf{y}} = \mathbf{y}^*} (\bar{y}_k - y_k^*) \; + \; \mathscr{O}[(\bar{y}_k - y_k^*)(\bar{y}_l - y_l^*)]. \tag{S9}$$

Substituting this expression into eq. (S7) and using the definition of the covariance matrix: $C_{kl}^* = \langle (\bar{y}_k - y_k^*)(\bar{y}_l - y_l^*) \rangle$ [this result follows from eq. (S8)] we find, to first order,

$$\hat{\Delta}_{ab} = \sum_{k,l} \left. \frac{\partial \hat{\theta}_a}{\partial \bar{y}_k} \right|_{\bar{\mathbf{y}} = \mathbf{y}^*} C_{kl}^* \left. \frac{\partial \hat{\theta}_b}{\partial \bar{y}_l} \right|_{\bar{\mathbf{y}} = \mathbf{y}^*}. \tag{S10}$$

In order to obtain an explicit expression for $\partial \hat{\theta}_a / \partial \bar{y}_k$ we differentiate eq. (S2) with respect to $\bar{y}_k$. This yields

$$0 = \sum_b \hat{h}_{ab} \frac{\partial \hat{\theta}_b}{\partial \bar{y}_k} - 2 \sum_i \left. \frac{\partial f_i(\boldsymbol{\theta})}{\partial \theta_a} \right|_{\boldsymbol{\theta} = \hat{\boldsymbol{\theta}}} R_{ik} \tag{S11}$$

where we introduced

$$\hat{h}_{ab} = 2 \sum_{i,j} \left. \frac{\partial^2 f_i(\boldsymbol{\theta})}{\partial \theta_a \partial \theta_b} \right|_{\boldsymbol{\theta} = \hat{\boldsymbol{\theta}}} R_{ij} (f_j(\hat{\boldsymbol{\theta}}) - \bar{y}_j) + 2 \sum_{i,j} \left. \frac{\partial f_i(\boldsymbol{\theta})}{\partial \theta_a} \right|_{\boldsymbol{\theta} = \hat{\boldsymbol{\theta}}} R_{ij} \left. \frac{\partial f_j(\boldsymbol{\theta})}{\partial \theta_b} \right|_{\boldsymbol{\theta} = \hat{\boldsymbol{\theta}}}. \tag{S12}$$

Solving eq. (S11) we obtain:

$$\frac{\partial \hat{\theta}_a}{\partial \bar{y}_k} = 2 \sum_i \sum_b (\hat{\mathbf{h}}^{-1})_{ab} \left. \frac{\partial f_i(\boldsymbol{\theta})}{\partial \theta_b} \right|_{\boldsymbol{\theta} = \hat{\boldsymbol{\theta}}} R_{ik}, \tag{S13}$$

which when substituted into eq. (S10) yields the following expression for the covariance of the estimated parameter, $\hat{\boldsymbol{\theta}}$:

$$\hat{\Delta}_{ab} = \left(4 \sum_{c,d} \sum_{j,k,l,m} (\hat{\mathbf{h}}^{-1})_{ac} \left. \frac{\partial f_j(\boldsymbol{\theta})}{\partial \theta_c} \right|_{\boldsymbol{\theta} = \hat{\boldsymbol{\theta}}} R_{jk} C_{kl}^* R_{lm} \left. \frac{\partial f_m(\boldsymbol{\theta})}{\partial \theta_d} \right|_{\boldsymbol{\theta} = \hat{\boldsymbol{\theta}}} (\hat{\mathbf{h}}^{-1})_{db} \right)_{\bar{\mathbf{y}} = \mathbf{y}^*}. \tag{S14}$$

We finally replace all exact quantities above by the corresponding sample estimators (and use $\mathbf{C} = \mathbf{Q}/M$), giving the key result, eq. (4) in the main text. The replacement of exact ensemble averages by sample estimates introduces bias terms which, to first order, are proportional to $1/M$, where M is the number of trajectories, see section E.1. For WLS-ICE/WLS-ECE procedures, we find that the bias is in practice often negligible (see main text). Just as the parameter estimates $\hat{\theta}_a$ are typically biased, so will the quantity $\hat{\phi}_{ab}$ in eq. (4) in the main text also be, as it is a nonlinear function of sample estimates, see section E.1. This bias can be reduced using the jackknife procedure applied to $\hat{\phi}_{ab}$ (see section G).

B Review of previous fitting procedures

In this section we investigate the two previous ubiquitous χ^2 methods for model fitting, namely WLS-ECE (uncorrelated χ^2) fitting and CCM (correlated χ^2) fitting.

B.1 WLS-ECE fitting

The previous most common method of functional fitting to data is the "standard" weighted least squares (WLS-ECE in the main text) method (uncorrelated χ^2 fitting), which is reviewed in this section. In this method, one assumes that all fluctuations around mean values are uncorrelated.

B.1.1 General fit functions

In the WLS-ECE method one maximizes the probability for the function $f(T_i;\boldsymbol{\theta}) = f_i(\boldsymbol{\theta})$ to have a good fit to the data:

$$P(\bar{\mathbf{y}};\boldsymbol{\theta}) \propto \prod_{i=1}^{N} \exp\left(-\frac{1}{2}\frac{(\bar{y}_i - f_i(\boldsymbol{\theta}))^2}{\overline{\sigma}_i^2}\right). \tag{S15}$$

Note that this probability is a product over the observations, $\bar{\mathbf{y}}$, hence the data is assumed to be statistically *independent*. Within this assumption, the unbiased estimator of variance of the mean is

$$\overline{\sigma}_i^2 = \frac{1}{M}\frac{1}{M-1}\sum_{m=1}^{M}(y_i^{(m)} - \bar{y}_i)^2. \tag{S16}$$

Maximizing the probability P is equivalent to minimizing

$$\chi^2 = \sum_{i=1}^{N}\frac{(\bar{y}_i - f_i(\boldsymbol{\theta}))^2}{\overline{\sigma}_i^2}, \tag{S17}$$

from which we get estimated parameters $\hat{\boldsymbol{\theta}}$, by solving

$$\left.\frac{\partial\chi^2}{\partial\theta_a}\right|_{\boldsymbol{\theta}=\hat{\boldsymbol{\theta}}} = 0 = 2\sum_i \left.\frac{\partial f_i(\boldsymbol{\theta})}{\partial\theta_a}\right|_{\boldsymbol{\theta}=\hat{\boldsymbol{\theta}}} \frac{1}{\overline{\sigma}_i^2}(f_i(\hat{\boldsymbol{\theta}}) - \bar{y}_i). \tag{S18}$$

For χ^2 close to the estimated parameter set $\hat{\boldsymbol{\theta}}$ we have the Taylor expansion

$$\begin{aligned}\chi^2 = \chi^2|_{\hat{\boldsymbol{\theta}}} &+ \sum_{a=1}^{K}(\theta_a - \hat{\theta}_a)\left.\frac{\partial\chi^2}{\partial\theta_a}\right|_{\boldsymbol{\theta}=\hat{\boldsymbol{\theta}}} \\ &+ \frac{1}{2}\sum_{a,b=1}^{K}(\theta_a - \hat{\theta}_a)(\theta_b - \hat{\theta}_b)\left.\frac{\partial^2\chi^2}{\partial\theta_a\partial\theta_b}\right|_{\boldsymbol{\theta}=\hat{\boldsymbol{\theta}}},\end{aligned} \tag{S19}$$

which we can insert back into the expression for P, eq. (S15), to yield

$$P(\boldsymbol{\theta}) = W\exp\left(-\frac{1}{4}\sum_{a,b=1}^{K}\hat{\boldsymbol{H}}_{ab}(\theta_a - \hat{\theta}_a)(\theta_b - \hat{\theta}_b)\right), \tag{S20}$$

where W is a normalization constant and

$$\hat{H}_{ab} = \left.\frac{\partial^2\chi^2}{\partial\theta_a\partial\theta_b}\right|_{\boldsymbol{\theta}=\hat{\boldsymbol{\theta}}} \tag{S21}$$

is the Hessian matrix, and we used $\partial\chi^2/\partial\theta_a|_{\boldsymbol{\theta}=\hat{\boldsymbol{\theta}}} = 0$. From eq. (S20) we find that

$$\hat{\Delta}_{ab} \equiv \langle(\hat{\theta}_a - \theta_a^*)(\hat{\theta}_b - \theta_b^*)\rangle = 2(\hat{\boldsymbol{H}})^{-1}_{ab}, \tag{S22}$$

i.e., the inverse of the Hessian matrix determines the covariances of the estimated parameters.

B.1.2 Linear fit functions

For the case that the fit function is linear, i.e., $f_i(\theta_1) = \theta_1 T_i$, eq. (S18) can be solved analytically (Press *et al.*[3]). The same can be done for the variance, σ^2, in the estimated parameter. We have

$$\hat{\theta}_1 = \frac{\sum_i \bar{y}_i T_i/\overline{\sigma}_i^2}{\sum_i T_i^2/\overline{\sigma}_i^2} \tag{S23a}$$

$$\hat{\sigma}^2 = \hat{\Delta}_{11} = \frac{1}{\sum_i T_i^2/\overline{\sigma}_i^2}. \tag{S23b}$$

B.2 CCM fitting

In this section we review CCM (correlated chi-square method) fitting procedure.[2,4–6]

B.2.1 General fit functions

Where a WLS-ECE fit only makes use of the diagonal (variance) of the covariance matrix, CCM makes use of the full matrix, defined as in eq. (S39), where the diagonal will be the square of the standard error of the mean, $s_i^2 = \sigma_i^2/M$. The task of fitting a function $f(t_i;\boldsymbol{\theta})$, reduces to maximizing the probability which is taken as the multi-variate Gaussian:

$$P(\overline{\boldsymbol{y}};\boldsymbol{\theta}) = Z^{-1}\exp\left(-\frac{1}{2}(\overline{\boldsymbol{y}}-\boldsymbol{f}(\boldsymbol{\theta}))^T\overline{\boldsymbol{C}}^{-1}(\overline{\boldsymbol{y}}-\boldsymbol{f}(\boldsymbol{\theta}))\right), \tag{S24}$$

where (for a good fit: $\boldsymbol{y}^* \approx \boldsymbol{f}$) $\overline{\boldsymbol{C}} = \overline{\boldsymbol{Q}}/M$ can be estimated through eq. (3) in the main text, and the normalization constant $Z = (2\pi)^{N/2}\sqrt{\det(\overline{\boldsymbol{C}})}$,[1] $\overline{\boldsymbol{y}} = (\overline{y}_1,\ldots,\overline{y}_N)$, $\boldsymbol{f} = (f_1,\ldots,f_N)$, with $f_i = f(T_i;\boldsymbol{\theta})$, and $(\ldots)^T$ denotes transpose. For uncorrelated data the covariance matrix estimator, $\overline{\boldsymbol{C}}$, will be diagonal and eq. (S24) reduces to eq. (S15), and the WLS-ECE method is attained.

As for WLS-ECE, maximizing P is equivalent to minimizing the cost function

$$\chi^2 = (\overline{\boldsymbol{y}}-\boldsymbol{f}(\boldsymbol{\theta}))^T\overline{\boldsymbol{C}}^{-1}(\overline{\boldsymbol{y}}-\boldsymbol{f}(\boldsymbol{\theta})). \tag{S25}$$

Thus, we get our estimated parameters $\hat{\theta}_a$ $(a = 1,\ldots,K)$ by solving:

$$\begin{aligned}\frac{1}{2}\frac{\partial\chi^2}{\partial\theta_a}\bigg|_{\boldsymbol{\theta}=\hat{\boldsymbol{\theta}}} = 0 &= -\frac{1}{2}\frac{\partial\boldsymbol{f}}{\partial\theta_a}\bigg|_{\boldsymbol{\theta}=\hat{\boldsymbol{\theta}}}\overline{\boldsymbol{C}}^{-1}(\overline{\boldsymbol{y}}-\boldsymbol{f}(\hat{\boldsymbol{\theta}})) + (\overline{\boldsymbol{y}}-\boldsymbol{f}(\hat{\boldsymbol{\theta}}))\overline{\boldsymbol{C}}^{-1}\left(-\frac{1}{2}\frac{\partial\boldsymbol{f}}{\partial\theta_a}\bigg|_{\boldsymbol{\theta}=\hat{\boldsymbol{\theta}}}\right)\\ &= \frac{\partial\boldsymbol{f}}{\partial\theta_a}\bigg|_{\boldsymbol{\theta}=\hat{\boldsymbol{\theta}}}\overline{\boldsymbol{C}}^{-1}(\boldsymbol{f}(\hat{\boldsymbol{\theta}})-\overline{\boldsymbol{y}}),\end{aligned} \tag{S26}$$

where in the last step we used the symmetry property of $\overline{\boldsymbol{C}}$, i.e., that $\overline{C}_{ij} = \overline{C}_{ji}$.

The derivation of the covariance, Δ_{ab}, of the CCM estimated parameters, $\hat{\theta}_a$ follows along identical lines as for WLS-ECE (previous section). Hence, Δ_{ab} is given by eq. (S22) where $\hat{\theta}_a$ is now obtained by solving eq. (S26) (instead of solving eq. (S18) as for WLS).

We finally note that the CCM is a maximum likelihood estimation procedure "asymptotically". More precisely, if M is large enough so that $\overline{\boldsymbol{y}}$s are Gaussian by the multi-variate central limit theorem, if the fit is "good" in the sense that $\boldsymbol{y}^* \approx \boldsymbol{f}$, and if the errors on the estimated elements of the covariance matrix are negligible, then the CCM is a maximum likelihood estimation method.

B.2.2 Linear fit functions

For fitting a *linear* function, $f_i(\boldsymbol{\theta}) = \theta_1 T_i$, to data one can determine the minimum of the CCM χ^2 function, eq. (S25), analytically. In particular, such a fit function is of relevance for BM (section C.1). Eq. (S26) becomes

$$0 = \frac{1}{2}\frac{\partial\chi^2}{\partial\theta_1}\bigg|_{\theta_1=\theta_1^*} = (\overline{\boldsymbol{y}}-\theta_1^*\,\boldsymbol{T})^T\overline{\boldsymbol{C}}^{-1}\boldsymbol{T}. \tag{S27}$$

Taking the second derivative we get

$$\frac{\partial^2\chi^2}{\partial\theta_1^2}\bigg|_{\theta_1=\theta_1^*} = -\boldsymbol{T}^T\overline{\boldsymbol{C}}^{-1}\boldsymbol{T}. \tag{S28}$$

From these results, as well as using eq. (S21) and eq. (S22), we get the estimated value for the parameter θ_1 and its variance σ^2 as

$$\hat{\theta}_1 = \frac{\overline{\boldsymbol{y}}^T\overline{\boldsymbol{C}}^{-1}\boldsymbol{T}}{\boldsymbol{T}^T\overline{\boldsymbol{C}}^{-1}\boldsymbol{T}} \tag{S29a}$$

$$\hat{\sigma}^2 = \hat{\Delta}_{11} = \frac{1}{\boldsymbol{T}^T\overline{\boldsymbol{C}}^{-1}\boldsymbol{T}}. \tag{S29b}$$

C Prototypical model systems

In the main text we provide results for different parameter estimation procedures. As prototype systems we use four processes where the true parameter values are known, namely: (i) Brownian motion (BM), (ii) damped harmonic oscillation (DHO), (iii) fractional Brownian motion (FBM), and (iv) continuous time random walks (CTRW). For BM and CTRW in d spatial dimensions, steps in different directions are independent. Therefore, without loss of generality, all simulations are here performed in one dimension, $d=1$, for these systems. Also, for consistency, we use $d=1$ in our FBM simulations.

C.1 Brownian motion

Our first example is a simple BM, which can be used to describe, e.g., single particle diffusion in one dimension. The mean square displacement (MSD) at time t, for dimension d, and diffusion constant D, is

$$\langle(\boldsymbol{x}(t)-\boldsymbol{x}(0))^2\rangle = \langle y(t)\rangle = \theta t, \tag{S30}$$

where

$$\theta = 2dD \tag{S31}$$

and

$$y(t) = [\boldsymbol{x}(t)-\boldsymbol{x}(0)]^2. \tag{S32}$$

In all simulations in the main text we use one-dimensional simulations, i.e., $d=1$.

In one-dimensional BM, the full covariance matrix for the displacement is known.[7] Choosing $x(0)=0$ and discretizing time into process times $t_i = i\varepsilon$ $(i=1,\dots,N)$, with time step ε, we have

$$V^*_{ij} = \langle(x_i-\langle x_i\rangle)(x_j-\langle x_j\rangle)\rangle = 2D\min(t_i,t_j), \tag{S33}$$

where $x_i = x(t_i)$ and D is the diffusion constant. On matrix form:

$$\boldsymbol{V}^* = 2D\varepsilon\begin{pmatrix} 1 & 1 & 1 & \dots & 1\\ 1 & 2 & 2 & & 2\\ 1 & 2 & 3 & & 3\\ \vdots & & & \ddots & \\ 1 & 2 & 3 & & N\end{pmatrix}. \tag{S34}$$

Of interest here is also the covariance matrix for the square displacements:

$$Q^*_{ij} = \langle(y_i-\langle y_i\rangle)(y_j-\langle y_j\rangle)\rangle. \tag{S35}$$

Using Wick's (Isserlis') theorem for zero-mean processes, we can calculate any moment of a multivariate Gaussian according to

$$\langle x_1x_2\cdots x_{2n}\rangle = \sum\prod\langle x_ix_j\rangle, \tag{S36}$$

where the sum is over all distinct ways of partitioning $x_1\dots,x_{2n}$ into pairs x_ix_j. Using eq. (S36) we have the following relation between $\boldsymbol{Q}^*$ and $\boldsymbol{V}^*$:

$$Q^*_{ij} = 2(V^*_{ij})^2. \tag{S37}$$

On matrix form:

$$\boldsymbol{Q}^* = 8(D\varepsilon)^2\begin{pmatrix} 1 & 1 & 1 & \dots & 1\\ 1 & 4 & 4 & & 4\\ 1 & 4 & 9 & & 9\\ \vdots & & & \ddots & \\ 1 & 4 & 9 & & N^2\end{pmatrix}. \tag{S38}$$

The standard unbiased sample estimator of $\boldsymbol{Q}$ is

$$\overline{Q}_{ij} = \frac{1}{M-1}\sum_m (y_i^{(m)}-\overline{y}_i)(y_j^{(m)}-\overline{y}_j). \tag{S39}$$

where m labels trajectories, see main text.

For BM, the inverse of the $\boldsymbol{Q}^*$ matrix is a tridiagonal matrix with column sum of zero, except the first. Explicitly

$$\boldsymbol{Q}^{*-1} = \frac{1}{8(D\varepsilon)^2} \begin{pmatrix} 1+\frac{1}{3} & -\frac{1}{3} & 0 & \dots & & \\ -\frac{1}{3} & \frac{1}{3}+\frac{1}{5} & -\frac{1}{5} & 0 & & \\ 0 & -\frac{1}{5} & \frac{1}{5}+\frac{1}{7} & -\frac{1}{7} & 0 & \\ \vdots & 0 & -\frac{1}{7} & \ddots & \ddots & \\ & & 0 & & & -\frac{1}{2N-1} \\ & & & & & \frac{1}{2N-1} \end{pmatrix}, \tag{S40}$$

which can be written as

$$(\boldsymbol{Q}^{*-1})_{ij} = \frac{1}{8(D\varepsilon)^2}\left[\left(\frac{1}{2i-1} + \frac{(1-\delta_{i,N})}{2i+1}\right)\delta_{i,j} - \left(\frac{1}{2i+1}\right)\delta_{i,j-1} - \left(\frac{1}{2i-1}\right)\delta_{i,j+1}\right]. \tag{S41}$$

where $\delta_{i,j}$ is the Kronecker delta-function ($\delta_{i,j}=1$, if $i=j$; $\delta_{i,j}=0$, if $i \neq j$). It is straightforward to show that indeed the matrix above satisfies $(\boldsymbol{Q}^{*-1}) \cdot \boldsymbol{Q}^* = \boldsymbol{I}$, where $\boldsymbol{I}$ is the identity matrix. Note that the results above for $\boldsymbol{Q}^{*-1}$ assumes that the time of the first sampling time is equal to the distance between subsequent sampling times. In general, this choice of sampling times may not be optimal. In such situations one can evaluate $\boldsymbol{Q}^{*-1}$ using numerical inversion of $\boldsymbol{Q}^*$ given in Eqs. (S37) and (S33).

C.2 Damped Harmonic Oscillation in a heat bath (DHO)

Following Nørrelykke and Flyvbjerg[8] we consider the dynamics of a damped harmonic oscillation in a heat bath (DHO). Physically, this process corresponds to the motion of a particle in a harmonic potential (i.e., the particle experiences a restoring force proportional to the displacement from the botttom of the potential) in a viscous liquid. Besides exerting friction on the particle, the molecules in the viscous liquid act as a noise source by providing thermal kicks on the particle. The equation of motion is:

$$m\frac{d^2x(t)}{dt^2} + \gamma\frac{dx(t)}{dt} + \kappa x(t) = F_{\text{therm}}(t), \tag{S42}$$

where $x(t)$ is the particle position at time t, m is the mass, γ is the friction constant, κ is the spring constant and $F_{\text{therm}} = (2k_BT\gamma)^{1/2}\eta(t)$ is the thermal noise, which is assumed to be zero mean Gaussian and delta-correlated, i.e.,

$$\langle \eta(t) \rangle = 0 \tag{S43}$$

and

$$\langle \eta(t)\eta(t') \rangle = \delta(t-t'). \tag{S44}$$

Above, k_B is the Boltzmann constant, T is the temperature of the heat bath and $\delta(z)$ is the Dirac delta-function. The equation of motion is completed by initial conditions for the position and velocity. We restrict ourself to

$$x(t=0) = x_0 \tag{S45}$$

$$v(t=0) = \left.\frac{dx(t)}{dt}\right|_{t=0} = 0, \tag{S46}$$

i.e., the particle is at the initial time displaced by a distance x_0 from its equilibrium position and then let go without imposing any initial velocity (no external pushing or pulling).

Based on eq. (S42) it is straightforward to derive an expression for the expected position, $\langle x(t) \rangle$, at time t. By taking the ensemble average of eq. (S42) and then making the ansatz: $\langle x(t) \rangle = \exp(i\Omega t)$ we arrive at a second order algebraic equation for Ω with two solutions:

$$\Omega_{\pm} = \frac{i}{2\tau} + \sqrt{\omega^2}, \tag{S47}$$

where

$$\tau = \frac{m}{\gamma}, \tag{S48}$$

$$\omega^2 = \omega_0^2 - \frac{1}{4\tau^2} \tag{S49}$$

and

$$\omega_0 = \sqrt{\frac{\kappa}{m}}. \tag{S50}$$

Thus, for the case $\omega^2 < 0$ the solution for $\langle x(t)\rangle$ is an exponentially damped function. For the case $\omega^2 > 0$, the solution is a complex valued exponential which can be written in terms of real-valued exponentials multiplied by sinus and cosinus functions. Also incorporating the initial conditions used here, eqs. (S45) and (S46), we find the solution for the mean to be

$$\langle x(t)\rangle = x_0\left(\cos(\omega t) + \frac{\theta_1}{\omega}\sin(\omega t)\right)\exp(-\theta_1 t), \tag{S51}$$

with

$$\theta_1 = \frac{1}{2\tau}. \tag{S52}$$

The case when $\omega = 0$ (i.e., $\omega_0 = 1/(2\tau)$) is referred to as critical damping. For this case we can obtain the solution from eq. (S51) by taking the limit of $\omega \to 0$ to find

$$\langle x(t)\rangle = x_0\,(1+\theta_1 t)\exp(-\theta_1 t). \tag{S53}$$

The case of critical damping is used in the simulations in the main text, where θ_1 is used as a fitting parameter.

Using the full stochastic eq. (S42), we can also derive an explicit expression for the covariance matrix $C^*(t,\tilde{t}) = \langle[x(t) - \langle x(t)\rangle][x(\tilde{t}) - \langle x(\tilde{t})\rangle]\rangle$. For simplicity we limit ourselves to the case $\omega^2 \geq 0$. We start by rewriting eq. (S42) as a set of two coupled first order equations[8]

$$\frac{d}{dt}\begin{pmatrix} x(t) \\ v(t) \end{pmatrix} = -\boldsymbol{M}\begin{pmatrix} x(t) \\ v(t) \end{pmatrix} + \begin{pmatrix} 0 \\ \frac{\sqrt{2D}}{\tau}\eta(t) \end{pmatrix}, \tag{S54}$$

with $D = k_B T/\gamma$ being the particle diffusion constant and

$$\boldsymbol{M} = \begin{pmatrix} 0 & -1 \\ \omega_0^2 & \frac{1}{\tau} \end{pmatrix}, \tag{S55}$$

which has the formal solution

$$\begin{pmatrix} x(t) \\ v(t) \end{pmatrix} = \begin{pmatrix} \langle x(t)\rangle \\ \langle v(t)\rangle \end{pmatrix} + \frac{\sqrt{2D}}{\tau}\int_0^t \exp(-\boldsymbol{M})(t-t')\begin{pmatrix} 0 \\ \eta(t') \end{pmatrix} dt', \tag{S56}$$

where

$$\begin{pmatrix} \langle x(t)\rangle \\ \langle v(t)\rangle \end{pmatrix} = \exp(-\boldsymbol{M}t)\begin{pmatrix} x_0 \\ v_0 \end{pmatrix} \tag{S57}$$

is the solution to the mean of eq. (S54) (using $\langle\eta(t)\rangle = 0$). The covariance matrix now becomes:

$$C^*(t,\tilde{t}) = \frac{2D}{\tau^2}\int_0^t dt' \int_0^{\tilde{t}} dt'' \left(\exp(-\boldsymbol{M}(t-t'))\right)_{12}\left(\exp(-\boldsymbol{M}(\tilde{t}-t''))\right)_{12}\langle\eta(t')\eta(t'')\rangle. \tag{S58}$$

Without loss of generality, we assume that $t < \tilde{t}$, and carry out the integral over t'' above to find:

$$C^*(t,\tilde{t}) = \frac{2D}{\tau^2}\int_0^t dt' \left(\exp(-\boldsymbol{M}(t-t'))\right)_{12}\left(\exp(-\boldsymbol{M}(\tilde{t}-t'))\right)_{12}. \tag{S59}$$

Using for $\omega^2 > 0$ the explicit form for the matrix exponential above as provided by Nørrelykke *et al.*[8]

$$\exp(-\boldsymbol{M}t) = \exp(-\theta_1 t)[\cos(\omega t)\boldsymbol{I} + \sin(\omega t)\boldsymbol{J}], \tag{S60}$$

with $\boldsymbol{I}$ the 2 by 2 identity matrix and

$$\boldsymbol{J} = \begin{pmatrix} \frac{\theta_1}{\omega} & \frac{1}{\omega} \\ -\frac{\omega_0^2}{\omega} & -\frac{\theta_1}{\omega} \end{pmatrix}, \tag{S61}$$

eq. (S59) becomes:

$$C^*(t,\tilde{t}) = \frac{8D\theta_1^2}{\omega^2}\int_0^t dt' \exp(-\theta_1(t-t'))\exp(-\theta_1(\tilde{t}-t'))\sin[\omega(t-t')]\sin[\omega(\tilde{t}-t')]. \tag{S62}$$

Carrying out the integral above we arrive at our final expression for the covariance matrix for DHO:

$$\begin{aligned} C^*(t,\tilde{t}) &= \frac{2D\theta_1}{\omega(\theta_1^2+\omega^2)}\exp(-\theta_1|\tilde{t}-t|)\{\omega\cos[\omega(\tilde{t}-t)]+\theta_1\sin[\omega|\tilde{t}-t|]\} \\ &+\frac{2D\theta_1^2}{\omega^2}\exp(-\theta_1(\tilde{t}+t))\left(\frac{\theta_1}{\theta_1^2+\omega^2}\cos[\omega(\tilde{t}+t)]-\frac{1}{\theta_1}\cos[\omega(\tilde{t}-t)]-\frac{\omega}{\theta_1^2+\omega^2}\sin[\omega(\tilde{t}+t)]\right). \end{aligned} \tag{S63}$$

In the limit $\omega \to 0$ (critical damping) we have

$$C^*(t,\tilde{t}) = \frac{2D}{\theta_1}\left(\exp(-\theta_1(\tilde{t}-t))(1+\theta_1(\tilde{t}-t)) - \exp(-\theta_1(\tilde{t}+t))\left(1+\theta_1(\tilde{t}+t)+2\theta_1^2\tilde{t}t\right)\right). \tag{S64}$$

To arrive at this result we made a Taylor series expansion to second order in ω^2 of the general expression.

We refrain from attempting to obtain an analytic expression for the inverse covariance matrix for DHO, as it appears a daunting task beyond the scope of the current study.

C.3 Fractional Brownian motion

Our third example is the case of one-dimensional FBM, which is a zero mean Gaussian process with autocorrelation function,[9]

$$v_{ij} = \langle x(t_i)x(t_j)\rangle = c(t_i^{2H}+t_j^{2H}-|t_i-t_j|^{2H}), \tag{S65}$$

at discrete times $t_i = i\varepsilon$ and where the parameter H denotes the Hurst parameter.[10] For $H = 1/2$, FBM becomes standard BM. Indeed, if we set $H = 1/2$ in eq. (S65) we find that $v_{ij} = c[(t_i+t_j)-|t_i-t_j|] = 2c\min(t_i,t_j)$ which is identical to eq. (S33) if we choose $c = D$. The inverse covariance matrix of eq. (S65) is (currently) not known analytically.

From eq. (S65) we get the MSD, for $t_i = t_j$, as ($x(0) = 0$)

$$\langle x^2(t)\rangle = \theta_1 t^{\theta_2}, \tag{S66}$$

where $\theta_1 = 2c$ and $\theta_2 = 2H$, i.e., the MSD has, for $H < 1/2$, a sublinear (or superlinear, if $H > 1/2$) dependence on time, t.

C.4 Continuous time random walk (CTRW)

Our last example uses CTRW in one dimension. Such a process is defined through a waiting time density $\psi(\tau)$, and a jump length probability density, $\zeta(\ell)$.[11] In our case we choose

$$\psi(\tau) = \frac{\alpha}{\tau^*}(1+\tau/\tau^*)^{-1-\alpha} \tag{S67}$$

with $0 < \alpha < 1$ so that we have infinite average waiting time $\langle\tau\rangle$. The jump length probability density is chosen to be a Gaussian:

$$\zeta(\ell) = \frac{1}{\sqrt{2\pi a^2}}\exp\left(-\frac{\ell^2}{2a^2}\right) \tag{S68}$$

with a variance a^2. For such a process, the MSD follows (for long times):[11]

$$\langle x(t)^2\rangle = \theta_1 t^{\theta_2} \tag{S69}$$

(with $x(0) = 0$) where

$$\theta_1 = \frac{2}{\Gamma(1+\alpha)\Gamma(1-\alpha)}\frac{a^2}{2(\tau^*)^\alpha}, \tag{S70}$$

and

$$\theta_2 = \alpha. \tag{S71}$$

D Simulation procedures

In this section we provide details about the methods used to generate the data for our prototypical example systems introduced in section C. Simulations ran to a stop time t_{stop}. All simulation parameters are listed in Sec. D.5.

D.1 Brownian motion (BM)

BM in one dimension is simulated using random jump lengths drawn from a normal distribution. In some detail, we start by taking the cumulative sum of N random numbers from a Gaussian distribution with zero mean and variance a^2, and square each element of the sum. Each step increments time by ε. This is repeated M times and summed and averaged. In short, the MSD was computed as:

$$\bar{y}_i = \frac{1}{M}\sum_{m=1}^{M}\left[\sum_{n=1}^{i} r_n^{(m)}\right]^2, \tag{S72}$$

where $r_n^{(m)}$ is a random number drawn from a normal distribution, associated with the length of the nth jump for trajectory m. The diffusion constant for this type of process is $D = a^2/(2\varepsilon)$.

D.2 Damped harmonic oscillation (DHO)

When simulating the harmonic oscillation in a heat bath, see eq. (S42), we follow the procedure described by Nørrelykke and Flyvbjerg[8] (at critical damping, $\omega = 0$).

D.3 Fractional Brownian motion (FBM)

For FBM simulations we used an algorithm by Davies and Harte.[12,13] When fitting the model in eq. (S66), we include only time points $t \geq T_1$ since this model prediction for the MSD, as for CTRW (see section D.4), is only valid for large simulation times.

D.4 Continuous time random walk (CTRW)

For generating the CTRW data we move a "particle" randomly with a step length drawn from a Gaussian probability density, eq. (S68), at each time step and increment time with a waiting time τ from the power-law distribution in eq. (S67). In more detail: while the process time, t, is smaller than the designated stop time we repeat the following procedure to generate one trajectory m:

1. Draw a random waiting time, τ, from the power-law in eq. (S67).
2. Move the particle, by increasing the current displacement by a random number r drawn from a normal distribution.
3. Update the time t by τ.

The procedure is repeated M times and averaged over, to yield the MSD. Since the prediction in eq. (S69) is only valid for $t \gg \tau^*$, for fitting purposes, we include only time points $t \geq T_1$ in the χ^2 expression, eq. (S1), and in the associated parameter covariance estimation formula, eq. (4) in the main text.

D.5 Simulation parameters

Below we list the simulation parameters used in all simulations in the main text and for the Supplementary Figures. We also give values for the first sampling time, T_1, used in the fit procedure (some of the functional forms used for fitting are only valid for "large" times).

- **BM.** Time increment, $\varepsilon = 1$ (dimensionless). Step length variance, $a^2 = 1$ (dimensionless). Simulation stop time $t = 10^4\varepsilon$. First sampling time, $T_1 = \varepsilon$.
- **DHO.** Spring constant $\kappa = 1$ (dimensionless). Mass $m = 1$ (dimensionless). Initial position, $x_0 = 1$ (dimensionless). Thermal energy, $k_BT = 10^{-2}$ (dimensionless). Simulation stop time, $t_{\text{stop}} = 20\omega_0^{-1}$ (with $\omega_0 = \sqrt{\kappa/m} = 1$). First sampling time, $T_1 = \omega_0^{-1}$.
- **FBM.** Hurst exponent, $H = 1/4$, unless stated otherwise. Time increment, $\varepsilon = 1$ (dimensionless). Prefactor in covariance matrix, $c = 1$ (dimensionless). Simulation stop time, $t_{\text{stop}} = 10^4\varepsilon$. First sampling time, $T_1 = 200\varepsilon$.
- **CTRW.** Power-law exponent, $\alpha = 0.5$. Step length variance, $a^2 = 1$ (dimensionless). Characteristic time scales $\tau^* = 1$ (dimensionless). Simulation stop time, $t_{\text{stop}} = 10^8\tau^*$. First sampling time, $T_1 = 10^5\tau^*$.

E Bias effects in parameter estimation

In this section, we provide analytical expressions for the bias in parameter (diffusion constant) estimation for BM. We find that for BM the CCM method has a bias which increases strongly with the number of sampling times, N. In contrast, the WLS method provides a (small) bias which is independent of N for large N. To make notation compact, we leave summations over repeated indices implicit (where no confusion can occur) in this section.

E.1 The origin of bias

In general the bias, i.e., the expected difference between some observable based on sample estimates and the "true" value of that observable, can be written as a series expansion in terms of $1/M$, where M is the number of trajectories.[14] To understand why this is so, in the present context, we recall that any sample mean or sample covariance, $\overline{Q}_{ijk....}$ (where i, j, k etc. labels sampling times), is an average (normalized sum) over the M trajectories. The multivariate central limit theorem tells us that for large M we can, for such averages, write $\overline{Q}_{ijk...} = Q^*_{ijk....} + \gamma_{ijk...}/\sqrt{M}$, where $\gamma_{ijk...}$ is a zero-mean "noise". Therefore any observable, O, which is a function of one, or several, such sample estimates (the optimal parameters $\hat{\boldsymbol{\theta}}$ and their associated covariance matrix $\hat{\boldsymbol{\Delta}}$, see previous sections, are examples of such observables) will (schematically) have a Taylor series expansion of the form:

$$O = O^* + \sum_{k=1}^{\infty} \frac{A_k}{\sqrt{M}M^{k-1}} + \sum_{k=1}^{\infty} \frac{B_k}{M^k} \tag{S73}$$

for large M. The first term in the Taylor expansion is the sought quantity, O^*. Considering the remaining terms, we note that, by construction, we have that $\langle A_1 \rangle = 0$, and hence the first non-zero term of the expectation value of the expression above is $\langle B_1 \rangle / M \propto 1/M$. For the case that the observable, O, is a function of more than one *independent* sample estimates, then we have $\langle A_k \rangle = 0$ for all k. However, note that if O is a function of several sample estimates which are *dependent*, then in general $\langle A_k \rangle \neq 0$ for $k \geq 2$. We can safely remove the first bias-term with a jackknife procedure,[15] see section G. Also higher order bias terms can be removed formally. However, already at the second order bias reduction level computational costs becomes considerable.

E.2 Bias in parameter estimation of CCM for linear fit functions

Consider equations (S1) and (S4). We write the sample estimator of the covariance matrix eq. (S39), and the exact, $\boldsymbol{Q}^*$, as related by

$$\overline{\boldsymbol{Q}}_{ij} = \boldsymbol{Q}^*_{ij} + \eta_{ij}, \tag{S74}$$

where $\boldsymbol{\eta}$ represents their deviation. We seek the "noise" in the inverse, $(\overline{\boldsymbol{Q}}^{-1})_{ij}$. Using the normalization condition, and writing

$$(\overline{\boldsymbol{Q}}^{-1})_{ij} = (\boldsymbol{Q}^{*-1})_{ij} + \xi_{ij}, \tag{S75}$$

we get

$$\boldsymbol{I} = \overline{\boldsymbol{Q}}\,\overline{\boldsymbol{Q}}^{-1} = (\boldsymbol{Q}^* + \boldsymbol{\eta})(\boldsymbol{Q}^{*-1} + \boldsymbol{\xi}) = \boldsymbol{I} + \boldsymbol{\eta}\boldsymbol{Q}^{*-1} + \boldsymbol{Q}^*\boldsymbol{\xi} + \boldsymbol{\eta}\boldsymbol{\xi}. \tag{S76}$$

Thus, to first order $\boldsymbol{\eta}\boldsymbol{Q}^{*-1} + \boldsymbol{Q}^*\boldsymbol{\xi} = 0$, and by definition $\boldsymbol{\eta} = \overline{\boldsymbol{Q}} - \boldsymbol{Q}^*$:

$$\boldsymbol{\xi} = \boldsymbol{Q}^{*-1} - \boldsymbol{Q}^{*-1}\overline{\boldsymbol{Q}}\,\boldsymbol{Q}^{*-1}. \tag{S77}$$

Using eq. (S75) in eq. (S1) and eq. (S4) yields

$$\begin{aligned} \hat{\theta} &= \frac{\bar{\boldsymbol{y}}^T(\boldsymbol{Q}^{*-1} + \boldsymbol{\xi})\boldsymbol{t}}{\boldsymbol{t}^T(\boldsymbol{Q}^{*-1} + \boldsymbol{\xi})\boldsymbol{t}} = \frac{\bar{\boldsymbol{y}}^T\boldsymbol{Q}^{*-1}\boldsymbol{t}}{\boldsymbol{t}^T\boldsymbol{Q}^{*-1}\boldsymbol{t}\left(1 + \frac{\boldsymbol{t}^T\boldsymbol{\xi}\boldsymbol{t}}{\boldsymbol{t}\boldsymbol{Q}^{*-1}\boldsymbol{t}}\right)} + \frac{\bar{\boldsymbol{y}}^T\boldsymbol{\xi}\boldsymbol{t}}{\boldsymbol{t}^T\boldsymbol{Q}^{*-1}\boldsymbol{t}\left(1 + \frac{\boldsymbol{t}^T\boldsymbol{\xi}\boldsymbol{t}}{\boldsymbol{t}\boldsymbol{Q}^{*-1}\boldsymbol{t}}\right)} \\ &\approx \frac{1}{\boldsymbol{t}^T\boldsymbol{Q}^{*-1}\boldsymbol{t}}\left(\bar{\boldsymbol{y}}^T\boldsymbol{Q}^{*-1}\boldsymbol{t} + \bar{\boldsymbol{y}}^T\boldsymbol{\xi}\boldsymbol{t} - \frac{\bar{\boldsymbol{y}}^T\boldsymbol{Q}^{*-1}\boldsymbol{t}}{\boldsymbol{t}\boldsymbol{Q}^{*-1}\boldsymbol{t}}\boldsymbol{t}\boldsymbol{\xi}\boldsymbol{t}\right), \end{aligned} \tag{S78}$$

where we did a series expansion to first order in $\boldsymbol{\xi}$. Using eq. (S77) we get

$$\begin{aligned} \hat{\theta} &= \frac{\bar{\boldsymbol{y}}^T\boldsymbol{Q}^{*-1}\boldsymbol{t}}{\boldsymbol{t}^T\boldsymbol{Q}^{*-1}\boldsymbol{t}} + \frac{\bar{\boldsymbol{y}}^T(\boldsymbol{Q}^{*-1} - \boldsymbol{Q}^{*-1}\,\overline{\boldsymbol{Q}}\,\boldsymbol{Q}^{*-1})\boldsymbol{t}}{\boldsymbol{t}^T\boldsymbol{Q}^{*-1}\boldsymbol{t}} - \frac{\bar{\boldsymbol{y}}^T\boldsymbol{Q}^{*-1}\boldsymbol{t}}{(\boldsymbol{t}^T\boldsymbol{Q}^{*-1}\boldsymbol{t})^2}\left(\boldsymbol{t}^T\boldsymbol{Q}^{*-1}\boldsymbol{t} - \boldsymbol{t}^T\boldsymbol{Q}^{*-1}\overline{\boldsymbol{Q}}\,\boldsymbol{Q}^{*-1}\boldsymbol{t}\right) \\ &= \frac{\bar{\boldsymbol{y}}^T\boldsymbol{Q}^{*-1}\boldsymbol{t}}{\boldsymbol{t}^T\boldsymbol{Q}^{*-1}\boldsymbol{t}} \underbrace{- \frac{\bar{\boldsymbol{y}}^T\boldsymbol{Q}^{*-1}\overline{\boldsymbol{Q}}\,\boldsymbol{Q}^{*-1}\boldsymbol{t}}{\boldsymbol{t}^T\boldsymbol{Q}^{*-1}\boldsymbol{t}} + \frac{\bar{\boldsymbol{y}}^T\boldsymbol{Q}^{*-1}\boldsymbol{t}}{(\boldsymbol{t}^T\boldsymbol{Q}^{*-1}\boldsymbol{t})^2}\boldsymbol{t}^T\boldsymbol{Q}^{*-1}\overline{\boldsymbol{Q}}\,\boldsymbol{Q}^{*-1}\boldsymbol{t}}_{\text{bias}=B}. \end{aligned} \tag{S79}$$

Note that the expectation value of the first term on the right hand side evaluates to θ^*, hence the additional terms yield the bias, whose expectation value, $\langle B \rangle$, we now seek. It is convenient to write eq. (S79) on component form (repeated indices are summed over) with $B = B_1 + B_2$ where

$$B_1 = -\frac{\overline{y}_k (\boldsymbol{Q}^{*-1})_{ki} \overline{Q}_{ij} (\boldsymbol{Q}^{*-1})_{jl} t_l}{\boldsymbol{t}^T \boldsymbol{Q}^{*-1} \boldsymbol{t}} \tag{S80a}$$

$$B_2 = \frac{\overline{y}_i (\boldsymbol{Q}^{*-1})_{ik} t_k t_j (\boldsymbol{Q}^{*-1})_{jm} \overline{Q}_{ml} (\boldsymbol{Q}^{*-1})_{ln} t_n}{(\boldsymbol{t}^T \boldsymbol{Q}^{*-1} \boldsymbol{t})^2} \tag{S80b}$$

(the component form of the quantity appearing in the denominators above is $\boldsymbol{t}^T \boldsymbol{Q}^{*-1} \boldsymbol{t} = t_p (\boldsymbol{Q}^{*-1})_{pq} t_q$). We thus see that the expected bias, $\langle B \rangle$, is determined by expectation value ($a, b, c \ldots$ label trajectories):

$$\langle \overline{y}_k \overline{Q}_{ij} \rangle = \frac{1}{M(M-1)} \langle \sum_{a=1}^{M} y_k^{(a)} \left[\sum_{b=1}^{M} y_i^{(b)} y_j^{(b)} - \frac{1}{M} \sum_{b=1}^{M} y_i^{(b)} \sum_{c=1}^{M} y_j^{(c)} \right] \rangle. \tag{S81}$$

E.3 Bias in parameter estimation of CCM for BM

Let us now consider the expected bias for CCM fitting for BM using the formal expression in section E.2. We have:

$$\langle y_k^{(a)} \rangle = \left\langle \left[x_k^{(a)} - x^{(a)}(0) \right]^2 \right\rangle = \sigma^{*2}_k = V^*_{kk}, \tag{S82}$$

where we in the last step used eq. (S33). Also $\langle x_i^{(a)} - x^{(a)}(0) \rangle = 0$, and since different realizations (trajectories) are independent we have

$$\langle x_i^{(a)} x_j^{(b)} \rangle = \delta_{a,b} V^*_{ij}. \tag{S83}$$

Higher order terms can be calculated using Wick's theorem, eq. (S36) (for large i, $x_i^{(a)}$ is a sum of many small increments, from the central limit theorem it follows that $x_i^{(a)}$ are Gaussian). We have

$$\begin{aligned} \langle y_i^{(a)} y_j^{(b)} \rangle &= \langle (x_i^{(a)})^2 (x_j^{(b)})^2 \rangle = \langle x_i^{(a)} x_i^{(a)} x_j^{(b)} x_j^{(b)} \rangle \\ &= \langle x_i^{(a)} x_i^{(a)} \rangle \langle x_j^{(b)} x_j^{(b)} \rangle + \langle x_i^{(a)} x_j^{(b)} \rangle \langle x_i^{(a)} x_j^{(b)} \rangle + \langle x_i^{(a)} x_j^{(b)} \rangle \langle x_i^{(a)} x_j^{(b)} \rangle \\ &= \sigma^{*2}_i \sigma^{*2}_j + 2 (V^*_{ij})^2 \delta_{a,b}. \end{aligned} \tag{S84}$$

Now, in the same way for higher order terms, we get

$$\begin{aligned} \langle y_k^{(a)} y_i^{(b)} y_j^{(c)} \rangle &= \langle x_k^{(a)} x_k^{(a)} x_i^{(b)} x_i^{(b)} x_j^{(c)} x_j^{(c)} \rangle = [\text{tedious enumeration of all cases}] = \\ &= \sigma^{*2}_k \sigma^{*2}_i \sigma^{*2}_j + 2\sigma^{*2}_k (V^*_{ij})^2 \delta_{b,c} + 2\sigma^{*2}_j (V^*_{ki})^2 \delta_{a,b} + 2\sigma^{*2}_i (V^*_{kj})^2 \delta_{a,c} + 8 (V^*_{ki})^2 (V^*_{kj})^2 (V^*_{ij})^2 \delta_{a,b} \delta_{b,c} \delta_{a,c}, \end{aligned} \tag{S85}$$

(no sum over repeated indices). Eq. (S81) now becomes

$$\langle \overline{y}_k \overline{Q}_{ij} \rangle = \frac{1}{M(M-1)} \underbrace{\sum_{a=1}^{M} \sum_{b=1}^{M} \langle y_k^{(a)} y_i^{(b)} y_j^{(b)} \rangle}_{U_1} - \frac{1}{M^2(M-1)} \underbrace{\sum_{a,b,c} \langle y_k^{(a)} y_i^{(b)} y_j^{(c)} \rangle}_{U_2}. \tag{S86}$$

Using eq. (S85) we get:

$$\begin{aligned} U_1 &= \sum_{a,b} \langle y_k^{(a)} y_i^{(b)} y_j^{(b)} \rangle \\ &= M^2 \sigma^{*2}_k \sigma^{*2}_i \sigma^{*2}_j + 2M^2 \sigma^{*2}_k (V^*_{ij})^2 + 2M \sigma^{*2}_j (V^*_{ki})^2 + 2M \sigma^{*2}_i (V^*_{kj})^2 + 8M V^*_{ki} V^*_{ij} V^*_{kj} \end{aligned} \tag{S87a}$$

$$\begin{aligned} U_2 &= \sum_{a,b,c} \langle y_k^{(a)} y_i^{(b)} y_j^{(c)} \rangle \\ &= M^3 \sigma^{*2}_k \sigma^{*2}_i \sigma^{*2}_j + 2M^2 \left[\sigma^{*2}_k (V^*_{ij})^2 + \sigma^{*2}_j (V^*_{ki})^2 + \sigma^{*2}_i (V^*_{kj})^2 \right] + 8M V^*_{ki} V^*_{ij} V^*_{kj}. \end{aligned} \tag{S87b}$$

Combining eq. (S87) with eq. (S86) results in:

$$\begin{aligned}\langle \bar{y}_k \overline{Q}_{ij} \rangle &= \frac{1}{M(M-1)} \left[(2M^2 - 2M) \sigma^{*2}_k (V^*_{ij})^2 + (8M - 8) V^*_{ki} V^*_{ij} V^*_{kj} \right] \\ &= 2\sigma^{*2}_k (V^*_{ij})^2 + \frac{8}{M} V^*_{ki} V^*_{ij} V^*_{kj}.\end{aligned} \tag{S88}$$

Using eq. (S88) in eq. (S80a) we find

$$\langle B_1 \rangle = -\frac{\sigma^{*2}_k (\boldsymbol{Q}^{*-1})_{ki} \delta_{i,l} t_l + \frac{8}{M} V^*_{ki} V^*_{ij} V^*_{kj} (\boldsymbol{Q}^{*-1})_{ki} (\boldsymbol{Q}^{*-1})_{jl} t_l}{\boldsymbol{t}^T \boldsymbol{Q}^{*-1} \boldsymbol{t}}, \tag{S89}$$

where we used that $Q^*_{ij} (\boldsymbol{Q}^{*-1})_{jl} = \delta_{i,l}$. Now consider B_2, eq. (S80b). We write eq. (S88) according to (also see eq. (S37))

$$\langle \bar{y}_i \overline{Q}_{ml} \rangle = \sigma^{*2}_i Q^*_{ml} + \frac{8}{M} V^*_{im} V^*_{ml} V^*_{li}. \tag{S90}$$

Eq. (S80b) now becomes

$$\begin{aligned}\langle B_2 \rangle &= \frac{(\boldsymbol{Q}^{*-1})_{ik} t_k t_j (\boldsymbol{Q}^{*-1})_{jm} \left[\sigma^{*2}_i Q^*_{ml} + \frac{8}{M} V^*_{im} V^*_{ml} V^*_{li} \right] (\boldsymbol{Q}^{*-1})_{ln} t_n}{(\boldsymbol{t}^T \boldsymbol{Q}^{*-1} \boldsymbol{t})^2} \\ &= \frac{\sigma^{*2}_i (\boldsymbol{Q}^{*-1})_{ik} t_k}{\boldsymbol{t}^T \boldsymbol{Q}^{*-1} \boldsymbol{t}} + \frac{8}{M} \frac{(\boldsymbol{Q}^{*-1})_{ik} t_k t_j (\boldsymbol{Q}^{*-1})_{jm} V^*_{im} V^*_{ml} V^*_{li} (\boldsymbol{Q}^{*-1})_{ln} t_n}{(\boldsymbol{t}^T \boldsymbol{Q}^{*-1} \boldsymbol{t})^2}.\end{aligned} \tag{S91}$$

Combining B_1 and B_2 we arrive at an expression for the predicted first order bias (eq. (S79)) for the suggested matrix, $\overline{\boldsymbol{R}}^{[CCM]}$; (notice the cancellations of the first terms):

$$\langle B \rangle = \frac{1}{M} \frac{8}{\boldsymbol{t}^T \boldsymbol{Q}^{*-1} \boldsymbol{t}} \left(\frac{(\boldsymbol{Q}^{*-1})_{ik} t_k t_j (\boldsymbol{Q}^{*-1})_{jm} V^*_{im} V^*_{ml} V^*_{li} (\boldsymbol{Q}^{*-1})_{ln} t_n}{\boldsymbol{t}^T \boldsymbol{Q}^{*-1} \boldsymbol{t}} - V^*_{ki} V^*_{ij} V^*_{jk} (\boldsymbol{Q}^{*-1})_{ki} (\boldsymbol{Q}^{*-1})_{jl} t_l \right), \tag{S92}$$

which can be analytically evaluated. With this in mind we use eq. (S33), with $t_i = i\varepsilon$, and eq. (S41), in eq. (S92). When evaluating the associated sums over repeated indices in eq. (S92), one uses:

$$\min(i,j) = \begin{cases} i, & \text{if } i \leq j \\ j, & \text{if } i > j \end{cases} \tag{S93}$$

and then splits the sums accordingly. This splitting leads to sums on the form

$$I(m,p) = \sum_k \frac{k^m}{(2k-1)^p}, \tag{S94}$$

where m and p are positive integers. These sums are rewritten according to

$$I(m,p) = \frac{1}{2^m} \sum_k \frac{1}{(2k-1)^p} \left((2k-1) + 1 \right)^m = \frac{1}{2^m} \sum_{q=1}^{m} \binom{m}{q} \sum_k (2k-1)^{q-p}, \tag{S95}$$

where we used the binomial theorem. The full calculation is tedious but straightforward. The final result is:

$$\langle B \rangle = \frac{D}{M} G(N) \tag{S96a}$$

$$G(N) = -\frac{a}{d} + \frac{b}{d^2} \tag{S96b}$$

$$a = \frac{N}{2} + s_1 - \frac{s_2}{2} \tag{S96c}$$

$$b = \frac{1}{16}(3s_1 - s_3) \tag{S96d}$$

$$d = \frac{s_1}{8} \tag{S96e}$$

$$s_n = \sum_{k=1}^{N} \frac{1}{(2k-1)^n}. \tag{S96f}$$

E.3.1 Asymptotic expansion

Let us now investigate eq. (S96) for large N. To that end, we write s_n, defined above, according to

$$s_n = \sum_{k=1}^{N}\left(\frac{1}{(2k-1)^n} + \frac{1}{(2k)^n} - \frac{1}{(2k)^n}\right) = \sum_{k=1}^{2N} k^{-n} - \frac{1}{2^n}\sum_{k=1}^{N} k^{-n}. \tag{S97}$$

In eq. (S96), there are three sums, s_1, s_2 and s_3. Out of these sums, s_1 decays most slowly with N and hence this sum is the only one which needs to be kept for large N. From eq. (0.131) in Gradshteyn *et al.*[16] we have

$$\sum_{k=1}^{N}\frac{1}{k} = \gamma + \ln N + \frac{1}{2N} + \mathscr{O}(\frac{1}{N^2}), \tag{S98}$$

where $\gamma \approx 0.5772$ is the Euler-Mascheroni constant. Combining the result above with eq. (S97) and eq. (S96) we arrive at the asymptotic expression

$$G(N) \approx -\frac{8N}{\ln N + \gamma + 2\ln 2}, \tag{S99}$$

where we used $\ln ab = \ln a + \ln b$. For large N, eq. (S99) is a good approximation compared to the exact bias, eq. (S96), see Supplementary Figure S3.

E.4 Bias in parameter estimation of WLS for BM

Let us now consider the second case, eq. (S5), of choosing $\boldsymbol{R}$. According to eqs. (S3) and (S5) we have the following:

$$\hat{\theta} = \frac{\overline{\boldsymbol{y}}^T \overline{\boldsymbol{Q}}_{\text{new}}^{-1}\boldsymbol{t}}{\boldsymbol{t}^T \overline{\boldsymbol{Q}}_{\text{new}}^{-1}\boldsymbol{t}}, \tag{S100}$$

where

$$\overline{Q}_{\text{new},ij} = \overline{Q}_{ij}\delta_{i,j} \tag{S101}$$

$$Q^*_{\text{new},ij} = Q^*_{ij}\delta_{i,j} \tag{S102}$$

$$(\overline{\boldsymbol{Q}}_{\text{new}}^{-1})_{ij} = \delta_{i,j}/\overline{Q}_{ij} \tag{S103}$$

$$(\boldsymbol{Q}^{*-1}_{\text{new}})_{ij} = \delta_{i,j}/Q^*_{ij}. \tag{S104}$$

The calculation starting from eq. (S77) to eq. (S79) is identical to before, just replace $\overline{\boldsymbol{Q}}$ with $\overline{\boldsymbol{Q}}_{\text{new}}$, and same for exact results. Since our new matrices are diagonal, eq. (S80) becomes (we here reintroduce explicit sums for the sake of clarity)

$$B_1 = -\frac{\sum_k \overline{y}_k \frac{1}{(Q^*_{kk})^2}\overline{Q}_{kk} t_k}{\sum_q t_q^2/Q^*_{qq}} \tag{S105a}$$

$$B_2 = \frac{\sum_{j,k}\overline{y}_k \frac{1}{Q^*_{kk}} t_k \cdot t_j^2 \frac{1}{(Q^*_{jj})^2}\overline{Q}_{jj}}{\left(\sum_q t_q^2/Q^*_{qq}\right)^2}. \tag{S105b}$$

Also the calculation from eq. (S81) which leads up to eq. (S88) is identical. From eq. (S105) we see that we need

$$\langle \overline{y}_k \overline{Q}_{jj}\rangle = 2\sigma^{*2}_k (V^*_{jj})^2 + \frac{8}{M}(V^*_{kj})^2 V^*_{jj} \qquad (j,k \text{ fixed}), \tag{S106a}$$

$$\langle \overline{y}_k \overline{Q}_{kk}\rangle = 2\sigma^{*6}_k + \frac{8}{M}\sigma^{*6}_k \qquad (k \text{ fixed}). \tag{S106b}$$

Substituting eq. (S106b) into eq. (S105a), and using eq. (S37) $Q^*_{kk} = 2(V^*_{kk})^2 = 2\sigma^{*4}_k$, and $\sigma^{*2}_k = 2Dt_k$ we get (with sums explicitly written)

$$\begin{aligned}\langle B_1\rangle &= -\frac{\sum_k \frac{1}{(Q^*_{kk})^2}\left(2\sigma^{*6}_k + \frac{8}{M}\sigma^{*6}_k\right) t_k}{\sum_q t_q^2/Q^*_{qq}} = -\frac{(1+\frac{4}{M})\sum_k 1/2D}{\sum_k 1/(2D)^2} \\ &= -2D\left(1+\frac{4}{M}\right).\end{aligned} \tag{S107}$$

In much the same way, we insert eq. (S106a) into eq. (S105b)

$$\begin{aligned}\langle B_2\rangle &= \frac{\sum_{j,k}\left(2\sigma^{*2}_k\sigma^{*4}_j+\frac{8}{M}\sigma^{*2}_j(V^*_{kj})^2\right)\frac{1}{2\sigma^{*4}_k}\frac{t_k t_j^2}{4\sigma^{*8}_j}}{\left(\sum_q t_q^2/2\sigma^{*4}_q\right)^2} = \frac{\sum_{j,k}\left(\frac{1}{4}\frac{1}{(2D)^3}+\frac{1}{M}\frac{1}{(2D)^5}\frac{(V^*_{kj})^2}{t_k t_j}\right)}{1/64D^4\left(\sum_k 1\right)^2} \\ &= 2D+\frac{2}{MD}\frac{1}{N^2}\underbrace{\sum_j\sum_k\frac{(V^*_{kj})^2}{t_k t_j}}_{I}.\end{aligned} \tag{S108}$$

Consider the double sum, I, in eq. (S108). We have time step $t_j=\varepsilon j$ and separate the sums into $j=k$ and $j\neq k$, which gives $V^*_{ij}=2D\varepsilon\min(i,j)$

$$\begin{aligned}I &= 4D^2\sum_{k=1}^{N}\sum_{j=1}^{N}\frac{[\min(j,k)]^2}{jk} = 4D^2\left(\sum_{k=1}^{N}1+2\sum_{k=1}^{N}\sum_{j=1}^{k-1}\frac{[\min(j,k)]^2}{jk}\right) \\ &= 4D^2\left(N+2\sum_{k=1}^{N}\frac{1}{k}\sum_{j=1}^{k-1}j\right) = 4D^2\left(N+2\sum_{k=1}^{N}\frac{1}{k}\frac{k(k-1)}{2}\right) \\ &= 4D^2\sum_{k=1}^{N}k = 2D^2N(N+1),\end{aligned} \tag{S109}$$

which inserted in eq. (S108) yields

$$\langle B_2\rangle = 2D+\frac{4D}{M}\left(\frac{1}{N}+1\right), \tag{S110}$$

from which we get the complete full bias together with eq. (S107):

$$\langle B\rangle = \langle B_1\rangle+\langle B_2\rangle = \frac{4D}{M}\left(\frac{1}{N}-1\right). \tag{S111}$$

Thus,

$$\boxed{\hat{\theta}-\theta^* = -\frac{4D}{M}\left(1-\frac{1}{N}\right).} \tag{S112}$$

Note that the bias is independent of N for large N.

E.5 Lack of bias for BMALS

We now consider our third and final choice of $\boldsymbol{R}$-matrix for BM. Since $\langle\bar{y}_i\rangle = y^*_i$ and $\boldsymbol{R}$ is a true inverse covariance matrix (and hence no sample estimate, see eq. (S6)) it follows immediately, by taking the expectation value of eq. (S3), that the BMALS parameter estimate is unbiased.

E.6 Lack of bias in parameter estimation of CCM for DHO

For the DHO problem we choose as our observable the particle position, i.e., we use $y_k^{(m)}=x_k^{(m)}$, where m labels different trajectories. For a good fit, the DHO parameter estimates $\hat{\boldsymbol{\theta}}$ are unbiased for CCM. To see this, consider the CCM minimization criterion eq. (S26) for DHO, which we write

$$0 = \left.\frac{\partial \boldsymbol{f}}{\partial\theta_a}\right|_{\boldsymbol{\theta}=\hat{\boldsymbol{\theta}}}\overline{\boldsymbol{Q}}^{-1}(\boldsymbol{f}(\hat{\boldsymbol{\theta}})-\boldsymbol{y}^*) - \left.\frac{\partial \boldsymbol{f}}{\partial\theta_a}\right|_{\boldsymbol{\theta}=\hat{\boldsymbol{\theta}}}\overline{\boldsymbol{Q}}^{-1}(\bar{\boldsymbol{y}}-\boldsymbol{y}^*). \tag{S113}$$

As in previous subsections, we then expand the inverse sample covariance matrix around its true value, i.e., we write $\overline{\boldsymbol{Q}}^{-1}=\boldsymbol{Q}^{*-1}+\boldsymbol{\xi}$, where $\boldsymbol{\xi}$ is given in eq. (S77). By expanding the right-hand side of eq. (S113) in $\boldsymbol{f}(\hat{\boldsymbol{\theta}})-\boldsymbol{y}^*$, $\bar{\boldsymbol{y}}-\boldsymbol{y}^*$ and $\boldsymbol{\xi}$, we arrive at

$$0 = \underbrace{\left.\frac{\partial \boldsymbol{f}}{\partial\theta_a}\right|_{\boldsymbol{\theta}=\hat{\boldsymbol{\theta}}}\boldsymbol{Q}^{*-1}(\boldsymbol{f}(\hat{\boldsymbol{\theta}})-\boldsymbol{y}^*)}_{\boldsymbol{F}(\hat{\boldsymbol{\theta}})} \tag{S114}$$

$$-\underbrace{\frac{\partial \boldsymbol{f}}{\partial \theta_a}\bigg|_{\boldsymbol{\theta}=\hat{\boldsymbol{\theta}}}\Big((\boldsymbol{Q}^{*-1}-\boldsymbol{Q}^{*-1}\overline{\boldsymbol{Q}}\,\boldsymbol{Q}^{*-1})(\boldsymbol{f}(\hat{\boldsymbol{\theta}})-\boldsymbol{y}^*)+\boldsymbol{Q}^{*-1}(\overline{\boldsymbol{y}}-\boldsymbol{y}^*)-(\boldsymbol{Q}^{*-1}-\boldsymbol{Q}^{*-1}\overline{\boldsymbol{Q}}\boldsymbol{Q}^{*-1})(\overline{\boldsymbol{y}}-\boldsymbol{y}^*)\Big)}_{\boldsymbol{G}(\hat{\boldsymbol{\theta}})}. \tag{S115}$$

Since $\boldsymbol{F}(\hat{\boldsymbol{\theta}})$ involves only the true covariance matrix and $\boldsymbol{y}^*$, the solution to $\boldsymbol{F}(\hat{\boldsymbol{\theta}})=0$ yields the true parameter value, i.e., we have $F_a(\boldsymbol{\theta}^*)=0$. If the fit is good, then we obtain the solution to eq. (S114) using a Taylor expansion, i.e., we write $F_a(\hat{\boldsymbol{\theta}})\approx F_a(\boldsymbol{\theta}^*)+\sum_b w_{ab}(\hat{\theta}_b-\theta_b^*)=\sum_b w_{ab}(\hat{\theta}_b-\theta_b^*)$, where $w_{ab}=\partial_b F_a(\hat{\boldsymbol{\theta}})/\partial\hat{\theta}_b|_{\hat{\boldsymbol{\theta}}=\boldsymbol{\theta}^*}$. Inserting this into eq. (S114) and solving for $\hat{\theta}_a$, we get

$$\hat{\theta}_a=\theta_a^*+\underbrace{\sum_b(\boldsymbol{w}^{-1})_{ab}G_b(\hat{\boldsymbol{\theta}})}_{B_a}. \tag{S116}$$

Thus, the bias in the estimated parameter, $\hat{\theta}_a$, is determined by the expectation value of B_a. An application of Wick's theorem for Gaussian variables yields

$$\langle\overline{y}_k\overline{Q}_{ij}\rangle=\langle\overline{y}_k\rangle Q_{ij}^*. \tag{S117}$$

This result is a direct consequence of the fact that the positions at different times for the DHO process are distributed according to a multivariate Gaussian. This Gaussianity, in turn, follows from the fact that the harmonic oscillator position is a linear function of the imposed Gaussian noise, see eq. (S42). Using eq. (S117) and the fact that $\langle\overline{y}_k\rangle=y_k^*$ and $\langle\overline{Q}_{ij}\rangle=Q_{ij}^*$ we find that $\langle\boldsymbol{G}(\hat{\boldsymbol{\theta}})\rangle=0$ and thereby that indeed $\langle B_a\rangle=0$, i.e., the CCM parameter estimate for DHO does not suffer from the bias problems discussed in the previous subsections.

F Approximate distribution for the estimated parameters

In the main text we saw that if M (the number of trajectories) is large enough the distribution for the estimated parameters is approximately Gaussian, see Figure 1 in the main text. To understand why this is so, we note that a set of random number, $\overline{y}_i$ ($i=1,\ldots N$), from the Gaussian distribution in eq. (S8) can be generated using

$$\overline{y}_i=y_i^*+\frac{1}{\sqrt{M}}\eta_i \tag{S118}$$

where $\boldsymbol{\eta}$ is a zero mean Gaussian random number with (M-independent) covariance matrix $\boldsymbol{Q}$. Consider now a function $F(\overline{\boldsymbol{y}})$, and note that the estimated parameters, θ_a, are functions of this type. We then Taylor-expand:

$$F(\overline{\boldsymbol{y}})\approx F(\boldsymbol{y}^*)+\frac{1}{\sqrt{M}}\boldsymbol{A}\cdot\boldsymbol{\eta}+O(\frac{1}{M}), \tag{S119}$$

where $\boldsymbol{A}$ is a matrix containing partial derivatives. Now assuming that the second term of the RHS above is non-zero, that the matrix A is full rank, and that all terms higher than or equal to $1/M$ can be neglected, we have that the distribution for F is another Gaussian. This follows from the fact that $\boldsymbol{A}\cdot\boldsymbol{\eta}$ is normally distributed if $\boldsymbol{\eta}$ are drawn from a multivariate Gaussian.[17]

G Jackknife bias reduction

Through data resampling, bias in data-fitting can often be reduced. Let O be the parameter estimator, based on some data set with M trajectories. The associated true parameter is denoted by O^*. Herein, we choose O as either the estimated parameters $\hat{\boldsymbol{\theta}}$, obtained by minimizing eq. (S1), or the associated covariance matrix $\hat{\boldsymbol{\phi}}$, eq. (4b) in the main text. As outlined in section E.1, one often expects such a finite data set to yield a bias contribution of the form

$$O=O^*+\frac{a}{M}+\frac{b}{M^2}+\frac{c}{M^3}+\mathcal{O}\left(\frac{1}{M^4}\right). \tag{S120}$$

The bias terms can be reduced by increasing the data samples, M, or by using the jackknife method.[15] Let us split the sample into g groups, each of size h, and define $O_{[-j]}$ as the parameter fitted to a data sample with the jth group removed.

G.1 First order jackknife bias reduction

The first order bias term can be removed through repeated fitting and averaging over the sampled data set:

$$O^{(1)} = \frac{1}{g}\sum_{j=1}^{g} O_{[-j]} \tag{S121a}$$

$$O_J^{(0,1)} = gO - (g-1)O^{(1)}. \tag{S121b}$$

By using eq. (S120) which has bias terms proportional to $M = hg$ for the full fitting, O, and $h(g-1)$ for the reduced sample estimator in eq. (S121), we see that we are left with

$$\begin{aligned} O_J^{(0,1)} &= O^* - \frac{b}{h^2}\frac{1}{g(g-1)} - \frac{c}{h^3}\left(\frac{1}{(g-1)^2} - \frac{1}{g^2}\right) + \mathscr{O}(g^{-3}) \\ &\approx O^* - \frac{b}{M^2} - 2\frac{c}{M^3}, \end{aligned} \tag{S122}$$

lacking the first order bias term. Although the higher order terms remain, their contribution is often lower than the first order term.

G.2 Second order jackknife bias reduction

For further bias reduction we can apply a second order correction. In a similar spirit to what is done in the first order jackknife, we split the data into g groups, and define $O_{[-j,-j']}$ as the parameter estimator based on a data set with the jth and j'th group removed, each of size h. Following Schucany *et al.*[18] we get

$$O^{(2)} = \frac{2}{g(g-1)}\sum_{j<j'}^{g} O_{[-j,-j']} \tag{S123a}$$

$$O_J^{(1,2)} = (g-1)O^{(1)} - (g-2)O^{(2)} \tag{S123b}$$

$$O_J^{(0,1,2)} = \frac{g}{2}O_J^{(0,1)} - \frac{g-2}{2}O_J^{(1,2)}. \tag{S123c}$$

If we combine our result with eq. (S120), we are only left with the third order term and the ones that follows it,

$$\begin{aligned} O_J^{(0,1,2)} &= O^* + \frac{c}{h^3}\frac{1}{g(g-1)(g-2)} + \mathscr{O}(g^{-4}) \\ &\approx O^* + \frac{c}{M^3}. \end{aligned} \tag{S124}$$

G.3 Variance for jackknife-bias-reduced estimators

In this section, we use eq. (S9) to show that $\hat{\theta}_a - \theta_a^*$ is insensitive (to lowest orders in $1/M$) to the jackknifing procedure. As a consequence, the covariance estimation formula, eq. (4) in the main text, remains valid also for jackknifed parameter estimations.

For later convenience, we define the derivative in eq. (S9) as

$$A_{a,i} = \left.\frac{\partial \hat{\theta}_a}{\partial \bar{y}_i}\right|_{\bar{\boldsymbol{y}}=\boldsymbol{y}^*}, \tag{S125}$$

which we will use in the following.

G.3.1 First order jackknife bias reduction

To first order the jackknife estimator is obtained by dividing the M trajectories into g groups of size h. Define the observable $\overline{O}_{[-j],i}$ as the estimate for observable O, in point i, with group j removed. In particular,

$$\bar{y}_{[-j],i} = \frac{1}{M-h}\sum_{m\neq m_j} y_i^{(m)} = \frac{1}{M-h}\left(\sum_{m=1}^{M} y_i^{(m)} - \sum_{m_j} y_i^{(m)}\right). \tag{S126}$$

The corresponding non-jackknifed estimator is

$$\bar{y}_i = \frac{1}{M}\sum_{m=1}^{M} y_i^{(m)}. \tag{S127}$$

The bias of the first order jackknife estimator of θ_a^* within the WLS-ICE method (see section A) is

$$\begin{aligned}
\theta_{J,a}^{(0,1)} - \theta_a^* =& g\hat{\theta}_a - (g-1)\left[\frac{1}{g}\sum_{j=1}^{g}\theta_{[-j],a}\right] - \theta_a^* = \frac{1}{h}\left[M\hat{\theta}_a - (M-h)\frac{1}{g}\sum_{j=1}^{g}\theta_{[-j],a}\right] \\
=& \frac{1}{h}\sum_i A_{a,i}\left(M(\overline{y}_i - y_i^*) - (M-h)\frac{1}{g}\sum_{j=1}^{g}\left(\overline{y}_{[-j],i} - y_i^*\right)\right) \\
=& \frac{1}{h}\sum_i A_{a,i}\left(\sum_{m=1}^{M}(y_i^{(m)} - y_i^*) - \frac{1}{g}\sum_{j=1}^{g}\left(\sum_{m=1}^{M}(y_i^{(m)} - y_i^*) - \sum_{m_j}(y_i^{(m_j)} - y_i^*)\right)\right) \\
=& \sum_i A_{a,i}\left(\frac{1}{gh}\sum_{j=1}^{g}\sum_{m_j}(y_i^{(m_j)} - y_i^*)\right) = \sum_i A_{a,i}\left(\frac{1}{M}\sum_{m=1}^{M}(y_i^{(m)} - y_i^*)\right) \\
=& \hat{\theta}_a - \theta_a^*,
\end{aligned} \tag{S128}$$

where we used eq. (S9) to get to the second and last (fifth) row, and eq. (S126)-(S127) for the third row. Thus

$$\theta_{J,a}^{(0,1)} - \theta_a^* = \hat{\theta}_a - \theta_a^*. \tag{S129}$$

Hence, jackknifing a parameter estimate does not change the (co)variance:

$$(\theta_{J,a}^{(0,1)} - \theta_a^*)(\theta_{J,b}^{(0,1)} - \theta_b^*) = (\hat{\theta}_a - \theta_a^*)(\hat{\theta}_b - \theta_b^*). \tag{S130}$$

G.3.2 Second order jackknife bias reduction

For the second order bias removal, the M trajectories are again divided into g groups. We define, as before, $\overline{O}_{[-j,-j'],i}$ as the estimate for observable O, in point i, with group j and j' removed. In particular

$$\overline{y}_{[-j,-j'],i} = \frac{1}{M-2h}\sum_{m\neq m_j, m_{j'}} y_i^{(m)} = \frac{1}{M-2h}\left(\sum_{m}^{M} y_i^{(m)} - \sum_{m_j} y_i^{(m_j)} - \sum_{m_{j'}} y_i^{(m_{j'})}\right). \tag{S131}$$

The average over all groups for θ_a is

$$\theta_a^{(2)} = \frac{1}{g(g-1)}\sum_{j\neq j'}\theta_{[-j,-j']}. \tag{S132}$$

The second order jackknife is now (as given by eq. (S123c))

$$\theta_{J,a}^{(0,1,2)} = \frac{g}{2}\theta_{J,a}^{(0,1)} - \frac{g-2}{2}\theta_{J,a}^{(1,2)}. \tag{S133}$$

Using eq. (S123b) we note

$$\begin{aligned}
\theta_{J,a}^{(1,2)} - \theta_a^* =& \frac{1}{h}\left((M-h)\left[\frac{1}{g}\sum_{j=1}^{g}\theta_{[-j],a}\right] - (M-2h)\left[\frac{1}{g(g-1)}\sum_{j\neq j'}\theta_{[-j,-j'],a}\right]\right) - \theta_a^* \\
=& \frac{1}{h}\sum_i A_{a,i}\left(\frac{1}{g}\sum_{j=1}^{g}\sum_{m=1}^{M}(y_i^{(m)} - y_i^*) - \frac{1}{g}\sum_{j=1}^{g}\left(\sum_{m_j} y_i^{(m_j)} - y_i^*\right)\right. \\
&\left. - \left[\frac{1}{g(g-1)}\sum_{j,j'}\sum_{m=1}^{M}(y_i^{(m)} - y_i^*) - \frac{1}{g(g-1)}\sum_{j,j'}\sum_{m_j}^{M}(y_i^{(m_j)} - y_i^*) - \frac{1}{g(g-1)}\sum_{j,j'}\sum_{m_{j'}}^{M}(y_i^{(m_{j'})} - y_i^*)\right]\right) \\
=& \frac{1}{h}\sum_i A_{a,i}\left(-\frac{1}{g}\sum_{j=1}^{g}\sum_{m_j}(y_i^{(m_j)} - y_i^*) + \frac{1}{g-1}\sum_{j'}\frac{1}{g}\sum_{j}\sum_{m_j}(y_i^{(m_j)} - y_i^*) + \frac{1}{g-1}\sum_{j}\frac{1}{g}\sum_{j'}(y_i^{(m_{j'})} - y_i^*)\right) \\
=& \frac{1}{h}\sum_i A_{a,i}\left(-\frac{1}{g} + \frac{1}{g} + \frac{1}{g}\right)\sum_j\sum_{m_j}(y_i^{(m_j)} - y_i^*).
\end{aligned} \tag{S134}$$

Thus

$$\theta_{J,a}^{(1,2)} - \theta_a^* = \sum_i A_{a,i} \frac{1}{M} \sum_j \sum_{m_j} (y_i^{(m_j)} - y_i^*) = \hat{\theta}_a - \theta_a^* \tag{S135}$$

and

$$(\theta_{J,a}^{(0,1,2)} - \theta_a^*) = \hat{\theta}_a - \theta_a^*. \tag{S136}$$

Thus the second order jackknife estimator has the same variance and covariance as non-jackknifed estimators.

H Estimation of errors on estimated parameters, using jackknife and bootstrap procedures

H.1 Jackknife error estimation

In the heuristic jackknife error estimation one makes use of the quantities $O_{[-j]}$, see section G, and calculates[19,20]

$$\sigma_J^2 = \frac{g-1}{g} \sum_{j=1}^{g} [O_{[-j]} - O^{(1)}]^2 \tag{S137}$$

where $O^{(1)}$ is given in eq. (S121). Then σ_J serves as an estimate for the error on the estimated parameter. Note that in contrast to jackknife *bias reduction* which is mathematically justified (based on the expected fluctuations around estimated mean values using the central limit theorem), there is in the general case no corresponding simple justification for the jackknife error estimation procedure for the present type of data.

H.2 Bootstrap error estimation

In the bootstrap error estimation, the scheme is:

- First, bootstrap[3,20,21] our original M trajectories, i.e., pick M trajectories from the original data *with replacement* (the same trajectory may be picked several times). Denote by $(\tilde{y}_i^{(m)}, t_i)$ the associated observables and compute the synthetic mean value of the chosen observable $\bar{y}_i = M^{-1} \sum_m \tilde{y}_i^{(m)}$.
- Make a weighted least squares (WLS) fit to the synthetic MSDs with respect to the fitting parameters. This fitting yields parameters $\tilde{\theta}_i$.

By repeating the two steps above many times (here, 100 times) we get a set of fit parameters $\tilde{\theta}_i$ $(i = 1, 2, \ldots, 100)$. From this set we simply compute the standard deviation as an estimator of the error for the fit parameters.[3,21]

I Coefficient of determination

We determine the goodness of fit by using the R^2 coefficient of determination, defined as

$$R^2 = 1 - \frac{S_{\text{res}}}{S_{\text{tot}}}. \tag{S138}$$

The method is based on a sum of squares over the N sampling points of, in our case, the mean positions or the MSD, $\bar{\mathbf{y}}$; hence, measuring the deviation from the sample mean in *time*,

$$\overline{Y} = \frac{1}{N} \sum_{i=1}^{N} \bar{y}_i \tag{S139}$$

$$S_{\text{tot}} = \sum_{i=1}^{N} (\bar{y}_i - \overline{Y})^2 \tag{S140}$$

$$S_{\text{res}} = \sum_{i=1}^{N} (f(t_i; \boldsymbol{\theta}) - \bar{y}_i)^2. \tag{S141}$$

Heuristically, a model that fits data perfectly has an $R^2 = 1$, while if it does not fit at all, $R^2 \ll 1$, see Supplementary Figure S5.

J Settings in "Particle Tracker" plug-in

For detecting and linking particles into trajectories from the Supplementary movies S1, S5 and S6 from the study by Chenouard et al.[22] we used the ImageJ plug-in "Particle Tracker"[23] (November 2016 version) with the following settings:

- 3D-data: no
- radius: 3
- cutoff: 3
- radius: 0.1
- LinkRange: 1 (default: 2)
- displacement: 10.00
- Dynamics: Brownian

and the following advanced options:

- Object features: 1.000
- dynamics: 1.000
- optimizer: greedy

All the settings listed above are default values except our choice for "LinkRange".

References

1. Van Kampen, N. G. *Stochastic processes in physics and chemistry*, vol. 1 Elsevier (1992).
2. Gottlieb, S., Liu, W., Renken, R. L., Sugar, R. L. & Toussaint, D. Hadron masses with two quark flavors. *Physical Review D* **38**, 2245–2265 (1988).
3. Press, W. H., Teukolsky, S. A., Vetterling, W. T. & Flannery, B. P. *Numerical Recipes 3rd Edition: The Art of Scientific Computing* (Cambridge University Press, 2007).
4. Van den Bos, A. *Parameter estimation for scientists and engineers* (John Wiley & Sons, 2007).
5. Seibert, D. Undesirable effects of covariance matrix techniques for error analysis. *Physical Review D* **49**, 6240–6243 (1994).
6. Michael, C. Fitting correlated data. *Physical Review D* **49**, 2616–2619 (1994).
7. Chaichian, M. & Demichev, A. Path integrals in physics, vol. 1: Stochastic processes and quantum mechanics. (IOP, Bristol, UK, 2001).
8. Nørrelykke, S. F. & Flyvbjerg, H. Harmonic oscillator in heat bath: Exact simulation of time-lapse-recorded data and exact analytical benchmark statistics. *Physical Review E* **83**, 041103 (2011).
9. Qian, H. Fractional Brownian motion and fractional Gaussian noise. In Processes with Long-Range Correlations, 22–33 (Springer, 2003).
10. Mandelbrot, B. B. & Van Ness, J. W. Fractional Brownian motions, fractional noises and applications. *SIAM Review* **10**, 422–437 (1968).
11. Metzler, R. & Klafter, J. The random walk's guide to anomalous diffusion: a fractional dynamics approach. *Physics Reports* **339**, 1–77 (2000).
12. Davies, R. B. & Harte, D. Tests for Hurst effect. *Biometrika* **74**, 95–101 (1987).
13. Chambers, M. The simulation of random vector time series with given spectrum. *Mathematical and Computer Modelling* **22**, 1–6 (1995).
14. Quenouille, M. H. Notes on bias in estimation. *Biometrika* **43**, 353–360 (1956).
15. Miller, R. G. The jackknife — a review. *Biometrika* **61**, 1–15 (1974).

16. Gradshteyn, I. & Ryzhik, I. Table of integrals, series and products (corrected and enlarged edition prepared by A. Jeffrey and D. Zwillinger). *Academic Press, New York* (2000).

17. Anderson, T. W. *An introduction to multivariate statistical analysis, 3rd ed.* (Wiley New York, 2003).

18. Schucany, W., Gray, H. & Owen, D. On bias reduction in estimation. *Journal of the American Statistical Association* **66**, 524–533 (1971).

19. Efron, B. & Stein, C. The jackknife estimate of variance. *The Annals of Statistics* 586–596 (1981).

20. Efron, B. & Tibshirani, R. J. *An introduction to the bootstrap* (CRC press, 1994).

21. Efron, B. & Tibshirani, R. Bootstrap methods for standard errors, confidence intervals, and other measures of statistical accuracy. *Statistical science* 54–75 (1986).

22. Chenouard, N. *et al.* Objective comparison of particle tracking methods. *Nature Methods* **11**, 281 (2014).

23. Sbalzarini, I. F. & Koumoutsakos, P. Particletracker (2016). http://imagej.net/Particle_Tracker. Version November 2016.
